# Defense strategies and associated phytohormonal regulation in *Brassica* plants in response to chewing and sap-sucking insects

**DOI:** 10.3389/fpls.2024.1376917

**Published:** 2024-04-05

**Authors:** Jamin Ali, Adil Tonğa, Tarikul Islam, Sajad Mir, Mohammad Mukarram, Alena Sliacka Konôpková, Rizhao Chen

**Affiliations:** ^1^College of Plant Protection, Jilin Agricultural University, Changchun, China; ^2^School of Life Sciences, Keele University, Newcastle-Under-Lyme, United Kingdom; ^3^Entomology Department, Diyarbakır Plant Protection Research Institute, Diyarbakir, Türkiye; ^4^Department of Entomology, Bangladesh Agricultural University, Mymensingh, Bangladesh; ^5^Department of Entomology, Rutgers University, New Brunswick, NJ, United States; ^6^Entomology Section, Sher-E-Kashmir University of Agricultural Science and Technology, Kashmir, India; ^7^Food and Plant Biology Group, Department of Plant Biology, Universidad de la República, Montevideo, Uruguay; ^8^Department of Integrated Forest and Landscape Protection, Faculty of Forestry, Technical University in Zvolen, Zvolen, Slovakia; ^9^Institute of Forest Ecology, Slovak Academy of Sciences, Zvolen, Slovakia

**Keywords:** herbivore feeding pattern, defense responses, Brassica, chewing herbivores, sap-sucking

## Abstract

Plants have evolved distinct defense strategies in response to a diverse range of chewing and sucking insect herbivory. While chewing insect herbivores, exemplified by caterpillars and beetles, cause visible tissue damage and induce jasmonic acid (JA)-mediated defense responses, sucking insects, such as aphids and whiteflies, delicately tap into the phloem sap and elicit salicylic acid (SA)-mediated defense responses. This review aims to highlight the specificity of defense strategies in *Brassica* plants and associated underlying molecular mechanisms when challenged by herbivorous insects from different feeding guilds (i.e., chewing and sucking insects). To establish such an understanding in *Brassica* plants, the typical defense responses were categorized into physical, chemical, and metabolic adjustments. Further, the impact of contrasting feeding patterns on *Brassica* is discussed in context to unique biochemical and molecular *modus operandi* that governs the resistance against chewing and sucking insect pests. Grasping these interactions is crucial to developing innovative and targeted pest management approaches to ensure ecosystem sustainability and *Brassica* productivity.

## 1 Introduction

The co-evolutionary arms race between plants and herbivorous insects has shaped the fascinating diversity of defense strategies observed in nature (Kareiva, 1999; Fürstenberg-Hägg et al., 2013). Plants have developed sophisticated defense responses to counter insect attacks, adapting to different herbivore feeding guilds. These defense responses are intricately connected to signaling pathways such as Jasmonic acid (JA), Salicylic Acid (SA), and Ethylene (ET) (War et al., 2012). These signaling pathways can regulate direct and indirect plant defense strategies that effectively deter, repel, and combat herbivorous insects (Kunkel and Brooks, 2002; Bari and Jones, 2009; Checker et al., 2018). The coordination of these defense pathways enables plants to deploy tailored and multifaceted responses, enhancing their ability to withstand and adapt to herbivores attack (Rejeb et al., 2014; Checker et al., 2018; Aftab and Roychoudhury, 2021).

*Brassica*, globally recognized as the second largest oilseed crop after soybean, holds a prominent position in the agricultural landscape (Attia et al., 2021). With an annual global production of around 72 million metric tons, this versatile crop plays a pivotal role in addressing food security, owing to its diverse uses, including oil extraction and as a crucial component in human diets (Mabry et al., 2021; Chen et al., 2023). However, *Brassica* crops face significant annual losses due to biotic stressors, particularly insect pests (Warwick, 2011; Baldwin et al., 2021). Approximately, 50-60% of *Brassica* crop production is believed to be susceptible to losses caused by insects and mites (Poveda et al., 2020). The worldwide pest management of crop plants including *Brassica* plants highly relies on insecticides which poses great environmental risks (Warwick, 2011). The susceptibility of *Brassica* crops to a multitude of insect pests and concerns regarding insecticide treatments underscore the pressing need for comprehensive understanding and the development of effective and sustainable control strategies to mitigate yield losses and safeguard its economic significance.

The plant immune system plays a pivotal role in shaping the dynamic interplay between plants and insect herbivores (Zhou and Zhang, 2020). As plants have evolved diverse defense strategies in response to varied feeding patterns of chewing and sucking insects, the importance of understanding these intricate molecular mechanisms requires specific attention (War et al., 2018). The ability of plants to discern and mount tailored defense responses, such as JA-mediated defenses against chewing insects and SA-mediated defenses against sucking insects, showcases the sophistication of their immune system (Nguyen et al., 2016; Stroud et al., 2022). Recognizing the specificity of these defense strategies is not only essential for comprehending plant-insect interactions but also holds immense significance for devising targeted pest management strategies (Vega-Álvarez et al., 2023). In the context of *Brassica* plants, where distinct physical, chemical, and metabolic adjustments contribute to defense (Ahuja et al., 2011), unravelling the intricacies of the plant immune system is key to developing innovative approaches that enhance ecosystem sustainability and ensure the productivity of *Brassica* crops.

This review primarily focuses on the interactions between herbivorous insects and plants belonging to the *Brassica* genus. Focusing on *Brassica*, we aim to provide a comprehensive synthesis of how chewing and sucking behaviors influence the activation of defense strategies mainly relying on involvement of JA and SA, the underlying molecular mechanisms (a specific focus on SA-JA crosstalk), and the impact on physical defense traits. Investigating *Brassica*’s responses, we highlight valuable insights into the broader mechanisms governing plant-insect interactions and defense strategies. In summary, this review will delve into the intricate interplay between herbivore feeding guilds and plant defense responses, with a specific emphasis on the unique attributes observed in *Brassica* species. By shedding light on the evolutionary trajectories of defense strategies, this study seeks to contribute to our understanding of the dynamic interactions between insects and plants in shaping the ecological landscape.

## 2 Defense responses and their mode of expression in *Brassica* against herbivorous insects

Plants have been coexisting with and facing endless challenges from herbivorous insects for hundreds of millions of years. Plants, including *Brassica* species, have evolved an arsenal of defense strategies to combat herbivore attack (Gatehouse, 2002; Ahuja et al., 2010). Plant defenses are broadly classified as direct and indirect defenses. Direct defenses are plant traits (e.g., trichomes, secondary metabolites) that reduce their susceptibility to insect herbivores or negatively affect insect biology or behavior (Chen, 2008; War et al., 2012). Indirect defenses are traits (e.g., herbivore-induced plant volatiles (HIPVs), extrafloral nectaries) that promote the attraction or efficacy of natural enemies of herbivorous insects such as predators and parasitoids (Heil, 2008; Aljbory and Chen, 2018). Both direct and indirect defenses can be expressed constitutively (i.e., always present in plants) or induced following insect attack. The metabolic costs of induced defenses are considered to be lower than constitutive defenses, particularly when insect pressure is sporadic (Karban, 2011), and there could be a trade-off between constitutive and induced defense responses (Zhang et al., 2008). Plant phytohormone signaling networks, particularly JA and SA signaling pathways play crucial roles in optimizing plant defenses against insect herbivores (Verma et al., 2016). In particular, the JA signaling cascade is considered a master regulator of induced plant responses to insect attack (Erb et al., 2012).

**Figure 1:**
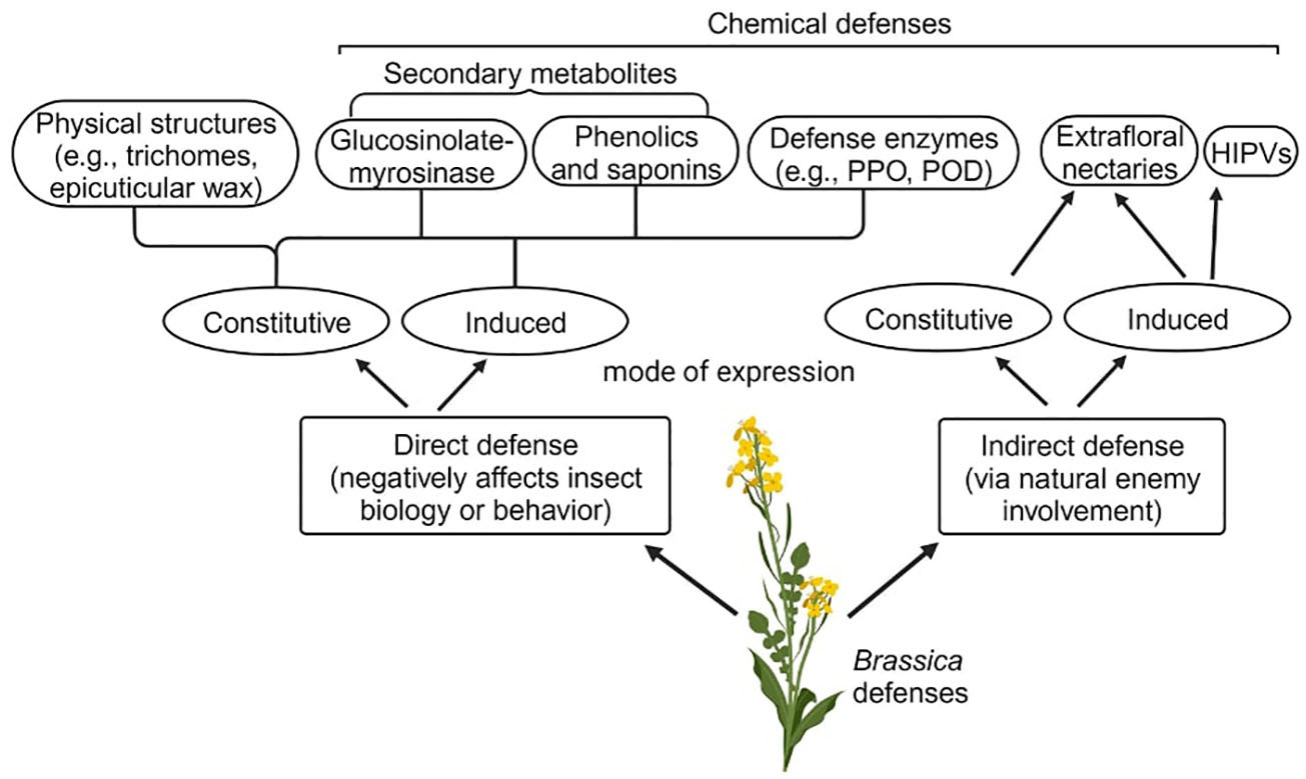
Outline of *Brassica* defenses against herbivorous insects. Both direct and indirect defenses can be expressed constitutively or induced following insect attack, or both. While physical defenses are typically expressed constitutively, certain physical defense structures such as trichomes and epicuticular wax could be induced in response to insect attack.

*Brassica* plants show a diverse array of direct physical and chemical defenses against herbivorous insects (**Figure 1**). Among physical defenses, epicuticular wax and trichomes account for one of the first lines of defenses against herbivores. For example, the presence of epicuticular wax was found to enhance *Brassica oleracea* resistance to the diamondback moth (*Plutella xylostella*), flea beetles (*Phyllotreta* spp.), and cabbage stink bugs (*Eurydema* spp.) (Bohinc et al., 2014; Silva et al., 2017). Although such morphological structures are constitutive defenses in *Brassica* plants, trichome density and epicuticular wax composition can be induced when challenged by insect herbivores (Traw and Dawson, 2002; Blenn et al., 2012).

The primary direct chemical defense in *Brassica* is the production of nitrogen- and sulfur-containing secondary metabolites known as glucosinolates (GS) that negatively affect insect herbivores (Hopkins et al., 2009; Jeschke et al., 2021), specifically generalist insects such as *Spodoptera littoralis* and *Mamestra brassicae* (Jeschke et al., 2017). GS are diverse in their structures (i.e., more than 130 known compounds) and are expressed constitutively in *Brassica* (Hopkins et al., 2009; Agerbirk and Olsen, 2012). The composition of GS in the family Brassicaceae varies depending on plant species, plant organs, ontogenetic stages, agricultural practices, and environmental conditions (Textor and Gershenzon, 2009; Ahuja et al., 2010). Although GSs per se could be toxic to insects (Kim et al., 2008), they become highly toxic when hydrolyzed by a specific enzyme called myrosinase and converted to toxic compounds such as isothiocyanates and nitriles (Agrawal and Kurashige, 2003; Wittstock et al., 2016). Both GSs and myrosinase are stored in adjacent but separate cells and GSs only encounter the enzyme when plant tissues are mechanically damaged by insect feeding (Hopkins et al., 2009).

Even though Brassicaceous plants possess GSs constitutively, their levels, particularly that of indole GSs, in tissues can be induced rapidly and substantially following shoot or root herbivory by insects (van Dam and Raaijmakers, 2006; Travers-Martin and Müller, 2007; Textor and Gershenzon, 2009). Insect attack can cause a redistribution of GSs in different organs or *de novo* synthesis of GSs in both attacked (i.e., local induction) and non-attacked (i.e., systemic induction) tissues (Hopkins et al., 2009; Touw et al., 2020). Likewise, the levels of myrosinase enzyme in plant tissues might increase upon insect feeding in some cases (Pontoppidan et al., 2003; Travers-Martin and Müller, 2007; Cachapa et al., 2021), although the impacts of such induction on plant defenses remain uncertain (Textor and Gershenzon, 2009).

Considering that some specialist herbivores such as *Pieris rapae* and *P. xylostella* can neutralize GS (Ratzka et al., 2002; Wittstock et al., 2004), other secondary metabolites such as phenolic compounds (e.g., flavonoids) and terpenoids (e.g., saponins) can confer direct resistance to specialist insects (Badenes-Perez et al., 2014; Ibrahim et al., 2018; Kovalikova et al., 2019). Moreover, cultivated *Brassica* plants can produce antioxidant defense enzymes such as polyphenol oxidase (PPO) and peroxidase (POD) and defensive proteins such as trypsin proteinase inhibitors (TPI) to defend specialist insects (Khattab, 2007; Ahmed et al., 2022). All these secondary metabolites and antioxidant enzymes can be present in *Brassica* constitutively or induced following insect attack, or both (Ibrahim et al., 2018; Kovalikova et al., 2019).

Brassicaceous plants produce herbivore-induced plant volatiles (HIPVs) when attacked by pest herbivores (**Figure 1**), including glucosinolate breakdown products such as nitriles and isothiocyanates (Uefune et al., 2012; Mathur et al., 2013c; Zhou and Jander, 2022). The emission of HIPVs can deter insect herbivores (Verheggen et al., 2013) and attract their natural enemies, thus facilitating the top-down control of herbivorous insects (Puente et al., 2008; Mathur et al., 2013c) Furthermore, *Brassica juncea* can produce extrafloral nectaries as an indirect defense, which can be present in plants constitutively, but the amount of nectar production could be induced following insect feeding (**Figure 1**) (Mathur et al., 2013a). The possession and induction of such nectaries could support natural enemies of herbivores by providing alternative foods (Jamont et al., 2013; Mathur et al., 2013a).

## 3 Impacts of herbivore feeding patterns on *Brassica* defense mechanisms

### 3.1 Jasmonic acid-mediated defenses in response to chewing insects

The majority of all known herbivorous insects belong to the orders, Coleoptera and Lepidoptera, that physically consume the plant tissues with their mouth parts evolved for chewing (Schoonhoven et al., 2005). For example, caterpillars follow a special pattern when feeding, removing uniform pieces of leaf tissue in a highly choreographed and predictable manner (Howe and Jander, 2008). Plants have developed various intricated mechanisms to perceive and respond to damage caused by chewing insects. The direct attack by chewing insects orchestrates a prompt and targeted host plant response, commencing a cascade of molecular events that yields in activation of JA-mediated plant defenses (Walling, 2000; Bari and Jones, 2009). Upon detection of chewing damage, plants release specific signals, such as damage-associated molecular patterns (DAMPs) and herbivore-associated molecular patterns (HAMPs) (Mithofer and Boland, 2008; Stahl et al., 2018; Uemura and Arimura, 2019). These signals are perceived by the plant receptors, triggering a signaling cascade that ultimately leads to the synthesis and accumulation of JA signaling.

As the core signaling pathway, JA is activated in response to herbivore chewing and wounding damage. The biosynthesis and signaling of JA have been reviewed elsewhere in detail (Hou and Tsuda, 2022; Kundu et al., 2023). Briefly, JA biosynthesis exist in various cellular compartments, primarily in the chloroplasts, peroxisomes, and cytosol (Staswick and Tiryaki, 2004; Erb et al., 2012; Wasternack and Hause, 2013). The precursor of JA biosynthesis is the unsaturated fatty acid linolenic acid (LA) which is derived from membrane phospholipids. Lipoxygenase (LOX) oxidize LA to 13-hydroperoxylinolenic acid (13-HPOT) which is afterward converted to 12-oxophytodienoic acid (OPDA) following two oxidation phases, allene oxide synthase (AOS) and allene oxide cyclase (AOC) (Ruan et al., 2019). Following the transportation of OPDA from chloroplast to the peroxisome, enzymatic reactions finally yield JA and its derivatives in cytosol (Erb et al., 2012; Ruan et al., 2019).

JA is perceived by F-box protein coronatine insensitive1 (COI1) which forms the SCF (Skp1-Cullin-F-box) E3 ubiquitin ligase complex, SCF^COI1^ (Yan et al., 2013). When JA is absent, jasmonate zim-domain (JAZ) proteins interact with transcription factors (TFs), repressing their activity (Katsir et al., 2008). For example, JAZ deficient mutants lead to increased expression of diverse transcript factors that yielded elevated resistance against the chewing insect *Trichoplusia ni* (Guo et al., 2018). JA-Ile binding to COI1 triggers the degradation of JAZ through the 26S proteasome (Ruan et al., 2019). This molecular cascade facilitates the activation of the transcription factor MYC2 and its homologs, culminating in the induction of JA-responsive genes harboring the G-box motif (CACATG) (Schweizer et al., 2013). Noteworthy participants in this regulatory network include auxiliary factors, exemplified by the mediator subunit MED25. Another significant transcription factor that is regulated by JAZ, Ethylene Insensitive 3 (EIN3) induces expression of ethylene-responsive TFs (ERFs) such as Octadecanoid-responsive AP2/ERF domain protein 59 (ORA59) (Vos et al., 2013). The MYCs constitute a significant group of TFs in response to chewing insects since they construct a mechanism that prioritize the responses to chewing damage and associated cues (Erb et al., 2012). The evidence clearly suggested that chewing insect feeding causes overexpression of MYC2 branch of JA pathway that activates JA-responsive genes, such as Vegetative Storage Protein 2 (VSP2) (Sheard et al., 2010). Furthermore, other TFs such as MYC3 and 4 interact with MYC2 and activate JA-mediated plant defense mechanisms against the damage by *S. littoralis* that induces JA accumulation in *Arabidopsis* (Schweizer et al., 2013; Schmiesing et al., 2016).

The genetic manipulation studies have revealed that several genes in JA signaling are overexpressed and play significant roles in response to chewing insects. For example, several LOX genes, *lox2*, *3*, *4*, and *6*, despite their distinct spatial expression, are induced upon wound damage (Wasternack and Hause, 2013). The *lox Arabidopsis* plants become severely susceptible to attack by *S. littoralis* feeding and artificial wounding with varying results for combinations of *lox* mutants (Glauser et al., 2008; Chauvin et al., 2013). Similarly, *AOS*-deficient *Arabidopsis* plants are susceptible to *S. littoralis* while *AOS*-overexpressed plants have enhanced resistance to this pest (Laudert et al., 2000). JA-mediated plant defenses against *S. littoralis* are completely impaired in *coi1 Arabidopsis* plants as well (Bodenhausen and Reymond, 2007). Similarly, *H*. *armigera* feeding was increased on *myc Arabidopsis* plants and decreased on MYC2-overexpressed *Arabidopsis* plants (Dombrecht et al., 2007). Other examples include the knockout of *JAR* and *JOX* genes, which results in an impaired JA signaling pathway that could not enhance resistance against wounding and *Mamestra brassicae*, respectively (Suza and Staswick, 2008; Caarls et al., 2017).

JA-mediated chemical defenses of *Brassica* plants include several classes of secondary metabolites such GSs, flavonoids, terpenoids, alkaloids, proteinase inhibitors (Howe and Jander, 2008). GSs are the predominant secondary metabolites present in *Brassica* plants, and most of the genes involved in GS biosynthesis are JA-inducible. The expression of these genes is governed by a functional regulatory module constituted by MYC and MYB TFs (Erb and Reymond, 2019). For instance, *Arabidopsis* feeding by *S. exigua* activates the JA pathway, resulting in an enhanced accumulation of GSs (Mewis et al., 2005). Notably, the genes participating in the biosynthetic pathway of GS are induced by JA, facilitated by the involvement of the bHLH TFs MYC2, MYC3, MYC4, and MYC5 (Yang et al., 2011; Schweizer et al., 2013). A coordinated functioning of MYB TFs is responsible for distinct branches of GSs biosynthesis, namely, methionine-derived aliphatic GS (MYB28, MYB29, and MYB76) and tryptophan-derived indole GS (MYB34, MYB51, and MYB122), which directly interact with MYC TFs, conferring resistance against *S*. *littoralis* (Gigolashvili, Berger, et al., 2007; Gigolashvili, Yatusevich, et al., 2007; Gigolashvili et al., 2008, 2009; Schweizer et al., 2013). The overexpression profile of MYB TFs such as MYB28 and MYB51 caused increased production of aliphatic and indole GS, respectively both of which adversely affected *S*. *exigua* (Gigolashvili, Berger, et al., 2007; Gigolashvili, Yatusevich, et al., 2007). In contrast, a double mutant *myb28 myb29* lacking aliphatic GS was more susceptible to the feeding activity by *Mamestra brassicae* (Beekwilder et al., 2008).

JA-mediated expression of different transcript factors can cause release of other secondary metabolites in addition to GSs. For example, *Arabidopsis* JAZ proteins interacting with bHLH TF MYB, regulate anthocyanin biosynthesis (Qi et al., 2011). In the presence of JA-Ile, the JAZ proteins are degraded, leading to the accumulation and overexpression of the WD-repeat–bHLH–MYB complex (Goossens et al., 2017). JA and its methyl esters MeJA are key elicitors of terpenoid indole alkaloid (TIA) biosynthesis. The key components of JA, including the JA co-receptor Coronatine Insensitive 1 (COI1) and the five JASMONATE ZIM-domain proteins CrJAZ1/2/3/8/10 have been characterized for their roles in regulating TIA biosynthesis (Patra et al., 2018). Terpenoids and GLVs often comprise a large and diverse portion of the volatile blends emitted by intact as well as damaged *Brassica* plants (van Poecke et al., 2001; Mumm et al., 2008).

Altogether, JA mediates various plant defenses against multiple attackers, especially herbivorous insects (Zhang et al., 2017). The biosynthetic pathways that lead to specialized metabolites especially secondary compounds such as terpenoids, alkaloids and GSs have been proven to be induced by the JA signaling pathway (De Geyter et al., 2012; Goossens et al., 2017). For example, *Arabidopsis* plants lacking GS biosynthesis responsive genes are highly susceptible to a wide range of chewing herbivores (Erb et al., 2012). *Arabidopsis fah1-7* deficient in the sinapoyl malate enzyme exhibits increased susceptibility to *P*. *brassicae* (Onkokesung et al., 2016), whereas reduced levels of kaempferol 3,7-dihamnoside in MYB75 overexpression lines correlate with increased *P. brassicae* performance.

JA defends plants indirectly by attracting natural enemies of insect pests through volatile emissions (Ozawa et al., 2008; Kappers et al., 2010). Parasitoids exhibit a keen ability to recognize HIPVs that are associated with their specific hosts and host plants. In specialized parasitoids, this ability may be innate (De Moraes et al., 1998), whereas generalist parasitoids learn to distinguish between different HIPV blends (Cardé and Bell, 1995). Natural enemies are responsive to common terpenoids, such as monoterpene (E)-ocimene and the monoterpene alcohol linalool (Dicke et al., 1990; Du et al., 1998), the methylene monoterpene (3E)-4,8-dimethyl-1,3,7-nonatriene, the methylene sesquiterpene (3E,7E)-4,8,12-dimethyl-1,3,7,11-tridectetraene (Dicke et al., 1990; Khan et al., 1997) and the sesquiterpene (E)-β-caryophyllene (Flint et al., 1979; Rasmann et al., 2005; Xiao et al., 2012). The emission levels of HIPVs are changed by *P. rapae* that attract parasitic wasps *Cotesia rubecula* (van Poecke et al., 2001; van Poecke and Dicke, 2002). The parasitism of *P*. *rapae* caterpillars by *C*. *rubecula* enhances plant fitness, increasing plant reproduction (van Loon et al., 2000). The perception ability of natural enemies of HIPVs emitting host plants may highly depend on survival strategy. For example, the specialist *Cotesia rubecula* can discriminate between induced host plants exposed to the damage by host larvae parasitized by conspecifics, while the generalist *C. glomerata* was unable to perform such a discrimination (Fatouros et al., 2005).

### 3.2 Salicylic acid-mediated defenses in response to sap-sucking insects

Sap-sucking insects encompassing a diverse array of pests, including aphids, whiteflies, thrips and so on, have a pivotal position in the functioning network of tropic levels. Sap-sucking insects exhibit distinct mouthpart morphology that are evolved based on their survival strategies. Several groups including aphids, mealybugs, psyllids and whiteflies search for a feeding site in the phloem veins, extending their stylets through cuticle, epidermis, and mesophyll (Walling, 2008). Thrips and mites suck the epidermal and mesophyll cell contents, puncturing using tube-like mouthparts while leafhoppers feed both on phloem and xylem contents (Mewis et al., 2005; Walling, 2008). The sap-sucking mouth parts do not cause a great damage on plant tissues by individual sap-suckers when compared with chewing insects while the sap-sucking damage may still have importance for plant immune system, especially when attacked by a settled population (Schoonhoven et al., 2005; Zhao et al., 2009). The significance of sap-sucking insects to plant immunity is not limited to tissue damage since an array of elicitors may accompany these pests while invading the host plants. Salivary, gut and honeydew endosymbiotic bacteria, salivary and ovipositional components, and associated plant pathogens such viruses may be involved in their attack on host plants (Wari et al., 2019). Once sap-sucking insects launch an attack on plant vascular tissues, plants induce SA-mediated defense responses.

SA is produced via two different signaling pathways; the isochorismate (IC) pathway, located in the chloroplast of plant cells and mediated by IC-synthase (ICS), and the phenylalanine ammonium (PA)-mediated by PA-lyase (PAL) pathways both of which are derived from chorismate (Dempsey et al., 2011). The core metabolite required for both signaling pathways is chorismate which is the main source of SA production in *Arabidopsis* (Wildermuth et al., 2001). *Arabidopsis* plants possess two ICS promoters, ICS1 and ICS2 which govern the chorismate-isochorismate conversion (Macaulay et al., 2017). The ICS1 promoter have WRKY and MYB TF binding sites which play roles in plant response against herbivores. These ICS enzymes individually or in combination can yield isochorismate (Strawn et al., 2007). IC amino acid conjugation producing isochorismate-9-glutamate, results in SA accumulation via avrPphB Susceptible3 (PBS3), a process exclusively characterized in *Arabidopsis*, followed either by spontaneous decomposition or enzymatic conversion via Enhanced Pseudomonas Susceptibility 1 (EPS1) (Jagadeeswaran et al., 2007; Nobuta et al., 2007; Rekhter et al., 2019; Torrens-Spence et al., 2019). The transportation of IC from chloroplast to cytosol requires Enhanced Disease Susceptibility 5 (EDS5) protein, a MATE transporter (Nawrath and Métraux, 1999; Nawrath et al., 2002). The studies with ICS mutants clearly revealed that SA can still be synthesized and accumulated. Further assessments including PAL-deficient plants have demonstrated that this SA biosynthesis in *Arabidopsis* continues via PAL pathway (Mauch-Mani and Slusarenko, 1996; Hu et al., 2010). However, there is a possible interplay between ICS- and PAL-mediated SA accumulation since a significant reduction was observed in ICS-mediated SA accumulation, when *Arabidopsis* plants lacked PAL activity. In PAL -mediated SA pathway, chorismate is converted to Phenylalanine that derivates *Trans*-cinnamic acid (t-CA) and then produces SA via benzoic acid (BA) via Abnormal Inflorescence Meristem 1 (AIM1) functioning (Dempsey et al., 2011).

**Figure 2:**
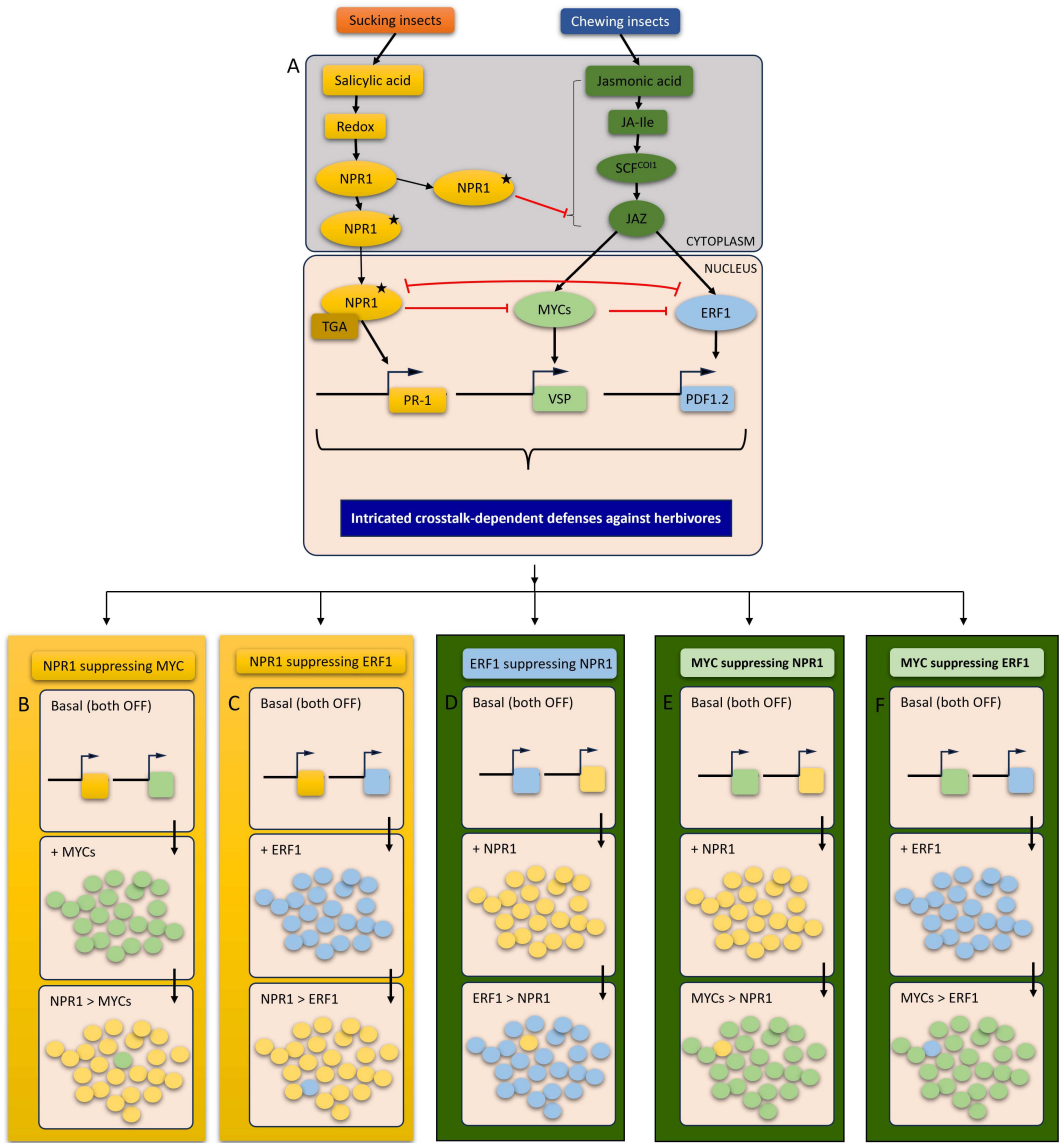
A schematic representation of SA-JA crosstalks in response to herbivory: **(A)** An overview of SA and JA induction in response to different feeding guilds depicting that chewing insects are more prone to induce JA-responsive plant defenses and sucking insects exhibits the tendency to trigger SA-responsive plant defenses. The overview of SA-JA cross-talk that is represented by red lines demonstrating the orientation of reciprocal suppression by respective transcription factors (TFs). The proposed models suggest two (cytosolic and nuclear) crosstalks steps between SA and JA via activated NPR1 (carrying a star). NPR1 activation occurs through the induction of a transition from an oligomeric state (NPR without star) to a monomeric state (with star), a prerequisite for its effective functioning. The activated NPR1 may participate directly in crosstalk, either within the cytoplasm or following translocation into the nucleus. The molecular consequences of possible crosstalk models as a function of reciprocal suppression effects of respective TFs. The side by side panels, **(B–F)** are consisted of three steps, first box showing the basal level of two TFs (boxes with two squares) which means plants are not under attack and the defense mechanisms are not induced, the second box showing the induced TF upon herbivory and the third box showing the suppressive effect of the antagonistic TFs. **(B)** indicates the suppression of MYC TFs by NPR1 that refers to SA-dependent plant responses are in control. **(C)** shows the suppression of ERF TFs by NPR1 that refers to SA-dependent plant responses are in control. **(D)** depicts the suppression of NPR1 by ERF1 and MYC, respectively, that refers to JA-dependent plant responses are in control. **(E)** shows the suppression of NPR1 by ERF1 and MYC, respectively, that refers to JA-dependent plant responses are in control. **(F)** depicts the crosstalk within JA signaling pathway which results in suppression of ERF1 by MYC. The arrows in **(B–F)** panels indicate the direction of working scheme of crosstalks between TFs. The circles in each box (under **(B–F)** panels) depicts the induction of respective TF while a single circle of suppressed TF is intentionally left in each crosstalk final to show the suppressive effect of the suppressor TF. The colors of squares and circles in **(B–F)** are based on the colors of TFs (NPR1, MYCs and ERF1 and respective genes) in panels **(A)** and **(B)** The background of panels **(B–F)** are based on SA and JA signaling pathways.

The involvement of SA signaling in plant defense systems could be either independent of or dependent on NPR1, a master regulator of plant defense mechanisms. In an NPR1-dependent manner, redox signals influence the activity of NPR1. For example, activated THIOREDOXIN h5 can reduce disulfide bonds in NPR1, causing monomerization and nuclear translocation of NPR1 (**Figure 2**) (Spoel et al., 2009). The *NPR1* was first identified in a screening of *Arabidopsis* mutants that were unable to activate the expression of *PR* genes or disease resistance (Cao et al., 1994; Delaney et al., 1994; Shah et al., 1997). The promoter region of the *NPR1* gene involves W-box sequences, which function as binding sites for WRKY family proteins. The mutations in the W-box sequences impair the expression levels of *NPR1* which underscores the significance of WRKY TFs in regulation of SA-NPR1 signaling (Yu et al., 2001). *NPR1* functions in two places namely the cytoplasm and the nucleus. The cytosolic NPR1 functioning is more related to its interplay with JA-responsive TFs, that finally yields their SA-JA crosstalk (**Figure 2A**), while nuclear *NPR1* is responsible for resistance development in response to stress factors. *NPR1* directly interacts with TGA TFs and NIMIN proteins. The TGA TFs directly interact with *PR-1* gene through binding to the activation sequence-1 (*as-1*) in its promoter region (Lebel et al., 1998). The requirement of SA for interactions between NPR1 and TGA TFs is highly TGA factor-specific. Interestingly, the presence of SA may also induce the expression of *NIMIN1*, *NIMIN2*, and *NIMIN3* genes while *NIMIN1* adversely affects SA-NPR1 signaling (Weigel et al., 2001, 2005). *NIMIN1* overexpression has a significant role which causes induction of ETI and SAR, while its reduced regulation enhances the induction of PR-1 gene by SA. *NPR1* is not always required for plant defenses. Transcription of several genes such as PR may require *NPR1*-independent SA signaling. The TFs responsible for SA-dependent and NPR1-independent resistance cover WHIRLY (WIH) and MYB genes. For example, SA can induce the single-stranded DNA binding activity of WHY, in an *NPR1*-independent manner (Desveaux et al., 2004). Furthermore, MYB30 positively regulates the pathogen-induced HR in an SA-dependent, NPR1-independent manner (Raffaele et al., 2006).

SA-mediated defense networks are interconnected, and the expression of certain genes, such as *ACD6, ALD1, PAD4, EDS1, EDS5, EPS1, ICS1/2, AIM1 PAL* and *PBS3/WIN3*, is inducible by SA, suggesting a mechanism of signal amplification involving both upstream and downstream components in the SA pathway. For example, feeding by *M. persicae* on *Arabidopsis* rosette leaves significantly induced the overexpression profiles of two genes: *NPR1*-dependent SA-associated genes *PR-1* and *BGL2* (Moran and Thompson, 2001). *Bemisia tabaci* feeding on *Arabidopsis* plants induced both local (*PR1, BGL2, PR5, SID2, EDS5, PAD4*) and systemic (*PR1, BGL2, PR5*) gene induction (Zarate et al., 2007).

The main secondary metabolite group in *Brassica* plants consists of GSs even when attacked by sap-sucking insects. For example, *M*. *persicae*-infested *Arabidopsis* plants release elevated levels of phenylpropanoid and isochorismate (van Poecke, 2007; Louis et al., 2012), which are highly dependent on and sensitive to the genes involved in SA biosynthesis. Furthermore, aphid infestation on *Arabidopsis* causes volatile derivatives of indolyl-GS and isothiocyanates (Mewis et al., 2005, 2006). Furthermore, feeding activity by *M*. *persicae* can cause *Arabidopsis* plants to release several terpenoids and the green leaf volatile, methyl salicylate (MESA) (Aharoni et al., 2003; van Poecke, 2007; Tholl and Lee, 2011).

The SA-mediated plant defenses are known to negatively affect the sap-sucking attackers while providing significant cues for foraging natural enemies. Methyl salicylate (MeSA), a volatile analogue of SA, attracts *Coccinella septempunctata* L. after infestation by the soybean aphid, *Aphis glycines* Matsumura (Zhu and Park, 2005). Salicylic acid analog, BTH (benzo-(1,2,3)-thiadiazole-7-carbothioic-acid S-methyl ester) enhances the suppression of *A*. *gossypii* without negative effects on the predatory larva *C*. *carnea* (Moreno-Delafuente et al., 2020).

### 3.3 The evolution of SA-JA crosstalk in response to herbivore feeding guilds

The induction of plant defense mechanisms commences upon perceiving the herbivore feeding and oviposition associated specific cues including DAMPs and MAMPs (Acevedo et al., 2015). Plants, upon recognition of these patterns, activate several intriguing signaling networks, including mitogen-activated protein kinase (MAPK) such as wound-induced protein kinase (WIPK), SA-induced protein kinase (SIPK) signaling cascades (Seo et al., 1995; Nürnberger and Scheel, 2001; Bonaventure, 2012; Acevedo et al., 2015). These signals are known to both positively or negatively regulate the defense-responsive phytohormones, JA and SA signaling pathways and corresponding downstream transcriptional responses (Jagodzik et al., 2018). These two signaling pathways are among the most significant pathways that are induced following herbivore attack and may frequently crosstalk.

The JA-SA crosstalk is reciprocally antagonistic in which the activation of one signaling pathway inhibits the counterpart (Thaler et al., 2012). This crosstalk is governed by the specific genes inherent to respective pathway that strategically disables the antagonist (Hickman et al., 2019). The crosstalk, as a clear advantage for plants, enhances the strategy of optimal energy and resource allocation for the most effective defense response, therefore, potentially plays a central role in the evolutionary regulation of plant defense mechanisms (Thaler et al., 2012). Therefore, plants have to first perceive the herbivores and associated cues and, afterward, develop the most suitable defense mechanism which are generally subject to crosstalks.

The establishment of defensive plant responses against insects highly depends on the phytohormonal signaling pathway and the regulative involvement of TFs that are central to crosstalks. The most commonly studied TFs involved in plant–insect interactions are MYCs, ERFs, MYBs, and WRKYs (**Figure 2A**). A growing body of evidence has demonstrated a clear suppression of both MYC and ERF branches of JA in SA-JA crosstalk. The well-known direct targets of JAZ repressors are closely related to bHLH factors, *MYC2*, *MYC3*, and *MYC4* (Fernández-Calvo et al., 2011). *AtMYC2*, for example, was reported to act downstream of JA and to regulate JA-dependent herbivore resistance (Dombrecht et al., 2007). These three MYCs, interacting with MYB proteins, regulate defense against insect herbivory by binding to a G-box motif found in the promoter of GS biosynthesis genes (Schweizer et al., 2013). The feeding damage of *P. rapae* on *Arabidopsis* plants induced JA pathway through the activation of the transcript factor, *MYC2* and JA-responsive marker gene, *VSP2* expression (**Figure 2B**) (De Vos et al., 2005; Verhage et al., 2011; Vos et al., 2015). Furthermore, the feeding of *P. rapae* on *Arabidopsis* strongly inhibited the other TFs of JA pathway, ERF-branch which includes the marker gene PDF1.2 (Verhage et al., 2011). The MYC2-branch including *VSP2* marker gene is known to regulate the defenses in response to wounding and chewing damage by insects while ERF-branch that covers high expression levels of PDF1.2 contributes plant defense in response to sucking insects (**Figure 2B**) (De Vos et al., 2005). In *Arabidopsis*, both of the JA-responsive genes *PDF1.2* and *VSP2* are highly sensitive to suppression by SA. Therefore, SA-dependent plant defense mechanisms suppresses both MYC and ERF branches of JA pathway (**Figures 2A, B**). WRKY TFs are considered to be responsible for the regulation of expression of *NPR1* and, accordingly, SA-dependent defenses (Bakshi and Oelmüller, 2014). For example, the overexpression of *WRKY70* enhances the expression of SA-responsive PR genes which plays suppressive roles against JA-responsive *PDF1.2*, the complete mechanism of which is *NPR1*-dependent (Li et al., 2004). Furthermore, the antagonistic effect of SA on JA signaling was shown to be controlled by NPR1 functioning in the cytosol (Spoel et al., 2003; Pieterse and Van Loon, 2004), with very recent findings indicating that *NPR1* physically interacts with *MYCs* for suppression of JA-responsive genes (**Figure 2A**) (Nomoto et al., 2021). The cytosolic NPR1 suppresses JA signaling in cooperation with other cytosolic factors such as *MPK4* and *PAD4* while nuclear *NPR1* suppresses *MYC2* (**Figure 2B**) (Pieterse et al., 2009; Nomoto et al., 2021).

The chewing insects have to overcome and manipulate the host plant for their own benefit so that they employ some other cues by activating the antagonistic signaling pathway of SA against JA. The compounds in salivary excretion of *S. exigua* namely, glucose oxidase (GOX), causes suppression of JA-regulated plant defense in *Arabidopsis* by activation of systemic acquired resistance (Weech et al., 2008). Furthermore, SA inhibited induced resistance of *Arabidopsis* in response to *S. exigua* through alteration of JA-dependent defense mechanisms such as defense protein activity and GS induction (Cipollini et al., 2004). Foliar treatment of *Arabidopsis* plants with egg extracts of two chewing herbivores, *P. brassicae* and *S. littoralis* significantly reduced the activation of several JA-responsive marker genes, the majority of which consists of MYC branch, and the employment of SA-deficient sid2-1 plants confirmed this suppression was controlled by SA (**Figure 2B**) (Bruessow et al., 2010). In comparison with chewing or wounding damage by *P. brassicae* and *S. littoralis* that induce accumulation of JA (Bruessow et al., 2010; Onkokesung et al., 2016), the involvement of egg-derived elicitors can cause a reversed induction of plant defenses through SA-JA crosstalk (Bruessow et al., 2010).

Interesting host manipulative engagements by insect pests with different feeding guilds covers a reversed version of crosstalk when compared to the case with chewing insects. These manipulative engagements suggest that JA-SA crosstalk may stem from the suppression of SA-responsive WRKY TFs by MYC branch of JA. For example, a previous study revealed the increased *B. brassicae* density with simultaneous *P. xylostella* infestation lowered the expression profile of WRKY and increased the expression profile of *MYC2* (**Figures 2A, B**) (Kroes et al., 2015). Furthermore, the infestation of *Brassica napus* plants with *B. brassicae*, exhibiting similar effects with JA treatments, had negative effects on the growth and development of the chewing pest, *P. xylostella* (Nouri-Ganbalani et al., 2018). The removal of the *COI1* receptor and *MYC* branch of JA resulted in a high-level accumulation of SA (Spoel and Dong, 2008). This manipulation is apparently not only for the benefit of the first attacker but also for the plant itself since they experience more intriguing defense responses. This seems quite phenomenal since a general understanding has suggested that JA mediates plant defenses upon feeding damage by chewing herbivores or artificial wounding and induces direct and indirect responses against the attacker and its natural enemies (Titarenko et al., 1997; Bruinsma et al., 2009).

In contrast to chewing insects, sap-sucking insect-induced plant defenses that are highly dependent on the attacker and feeding damage. For example, sap-sucking insect species associated with higher cell damage are more prone to induce JA-dependent plant responses while those with lower cell damage can induce SA dependent responses. For example, the higher level of JA-responsive marker gene, PDF1.2 and respective plant defense mechanisms of Arabidopsis in response to *B. brassicae* and *F. occidentalis* when compared with *M. persicae* is likely corresponded to relatively greater cell damage during the process of reaching the phloem as a function of distinct probing behavior (Cole, 1997; Moran et al., 2002; De Vos et al., 2005). For aphid species, *M. persicae*, the crosstalk seems more complex as such SA dependent plant responses with PR-1 and BGL-2 and JA-dependent responses with PDF1.2 and LOX2 constitute a simultaneous expression for both pathways while SA-responsive expression was dominative over JA-responsive marker genes (Moran and Thompson, 2001). However, further factors rather than cell damage can interfere with plant defense responses to sap-sucking insects. In Arabidopsis plants that suffered *Eurydema oleracea* feeding activity, *PDF1.2* gene expression was suppressed by the activation of *PR1a* and *ICS1* (Ederli et al., 2020). Therefore, an attack by *E. oleracea* clearly activates SA pathway and suppresses JA defenses (Costarelli et al., 2020). Similarly, in response to *B. tabaci*, the gene transcripts responsive to SA (*PR1*, *BGL2*, *PR5*, *SID2*, *EDS5*, *PAD4*) were activated while those responsive to both MYC2 and ERF branches of JA (*PDF1.2*, *VSP1*, *FAD2*, *FAD3*, *FAD7*, *THI2.1*, *COI1*) were either suppressed or non-respondent (Kempema et al., 2007; Zarate et al., 2007; Zhang et al., 2013).

In general, one signaling pathway is expected to suppress the other since a crosstalk between JA and SA prioritizes one signaling defense pathway over the other in response to herbivore attack. The expression of marker genes of both signaling pathway may be due to a concentration-dependent degree of crosstalk (Moran and Thompson, 2001; Mur et al., 2006; Spoel et al., 2007).

Insect attack could induce plant defense mechanisms other than JA and SA. For instance, *P. brassicae* egg deposition in *Arabidopsis* plants has been shown to cause localized cell death, callose accumulation, and the production of reactive oxygen species (Little et al., 2007). The induction of these defense mechanisms in response to oviposition-associated cues can manipulate host plants defenses for the benefit of the ovipositing pest, preventing other attackers (Orlovskis and Reymond, 2020). Plants facing antagonistic attackers may develop intricate defense systems that hinder their ability to respond effectively to secondary attackers. This complexity arises from the activation of signaling pathways by the primary attackers, rendering it challenging for plants to reversely crosstalk, while protecting the balance of resource allocation, and thereby rendering them more susceptible to subsequent assaults from secondary attackers (Vos et al., 2015). Whether this is the case depends highly on the concentrations and combinations of activated defensive proteins and VOCs upon triggered signaling pathway (Smith and Boyko, 2007; Howe and Jander, 2008).

### 3.4 Glucosinolate biosynthesis and regulation in *Brassica*

**Figure 3:**
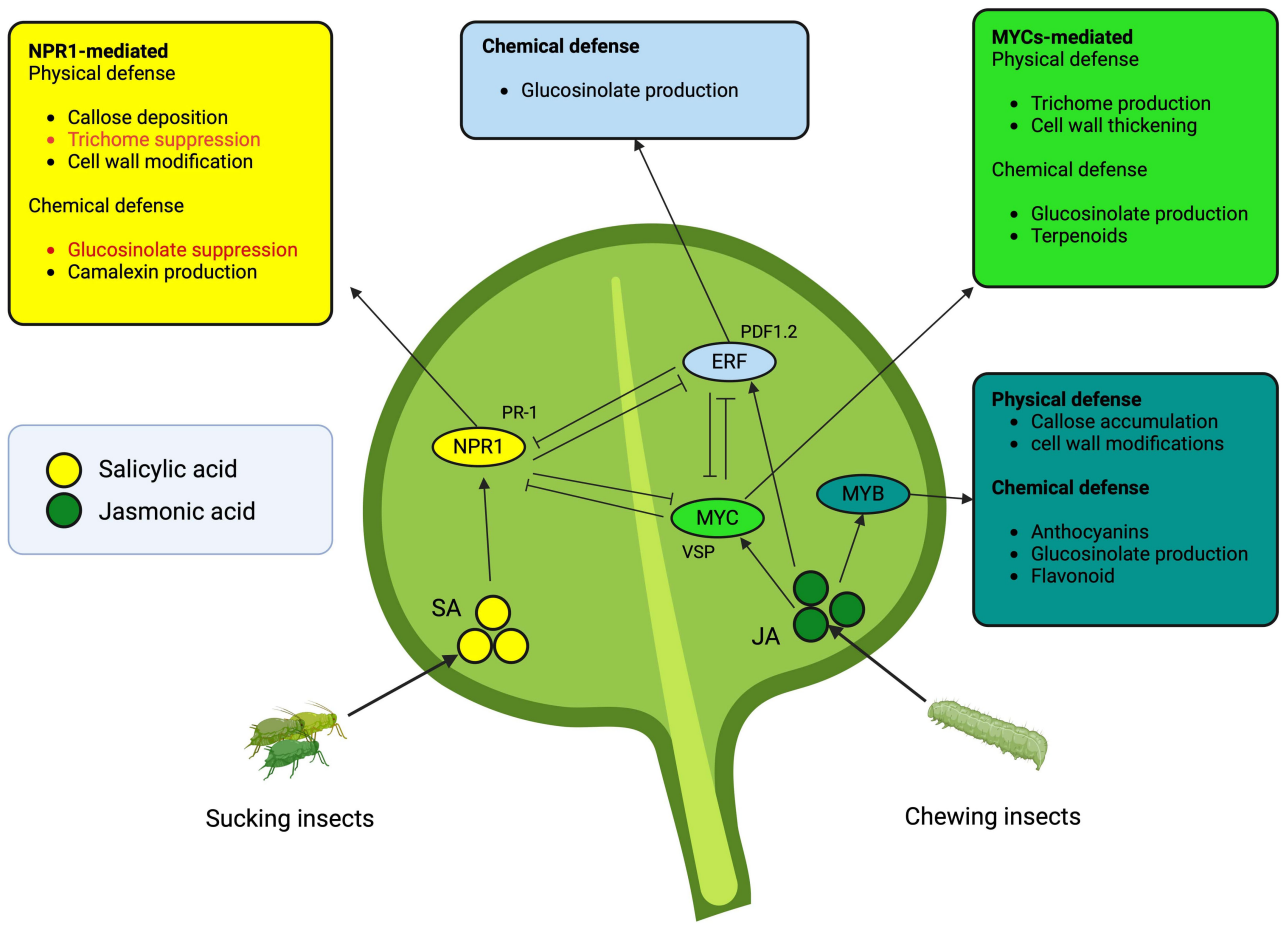
Salicylic acid- and jasmonic acid-mediated plant defense mechanisms are orchestrated by transcription factors (TFs). The color of each outer box corresponds to the ellipses indicating TFs. Red fonts depict suppression of the mechanisms, whereas the black font means accumulation or production of respective defense mechanism. The arrows indicate direct induction while inhibitory lines suggest negative crosstalks. The TFs in ellipses induce the upregulation of the gene group (most common) attached around.

Glucosinolates are pivotal plant defense compounds in *Brassica*, exhibiting structural and ecological diversity (Hopkins et al., 2009). The intricate regulatory network governing GS biosynthesis dynamically responds to stress, immune triggers, and herbivory, thereby influencing plant fitness (Zukalová and Vasak, 2002; Bruce, 2014; Mitreiter and Gigolashvili, 2021) (**Figure 3**). The evolutionary significance of GSs is underscored by the interplay of genes, TFs, and hormonal cues (Schweizer et al., 2013; Mitreiter and Gigolashvili, 2021). Subgroup 12 R2R3 MYB TFs (e.g., MYB28, MYB29) positively regulate GSs, forming complexes with bHLH proteins, while Subgroup IIIe bHLHs (e.g., MYC2, MYC3) modulate GS types in response to phytohormones like jasmonate (Gigolashvili et al., 2008; Seo and Kim, 2017; Millard et al., 2019). Hormonal interactions, especially the JA-SA crosstalk, highlight nuanced control mechanisms governing plant immunity and GS production (Tsuda et al., 2009; Guo et al., 2013). Upon plant damage, GSs initially biologically inert, become potent through myrosinase-driven hydrolysis, yielding compounds responsible for toxicity and herbivore deterrence (Bones and Rossiter, 1996). Over 130 GS structures exist, predominantly within *Brassica* (Newton et al., 2009; Textor and Gershenzon, 2009). Herbivory induces GS production, with indolic GSs showing a consistent 1.2- to 20-fold increase, irrespective of the herbivore type (Sontowski et al., 2019). The jasmonate signaling cascade activates TFs controlling GS biosynthesis, while the functions of myrosinase-associated proteins remain inadequately studied.

Insect herbivores from different feeding guilds can influence glucosinolate biosynthesis and regulation in plants (Hopkins et al., 2009; Textor and Gershenzon, 2009). Sucking herbivores, exemplified by aphids, generally induce fewer changes in GSs and associated gene expression compared to chewing insects like beetles and caterpillars (Barth and Jander, 2006; Sato et al., 2019). This distinction is rooted in aphids’ feeding behavior, targeting single phloem cells and spatially separating them from myrosinase, potentially avoiding the trigger for glucosinolate breakdown (Barth and Jander, 2006). Despite the typically low induction of GSs in response to sucking herbivores, recent investigations into *M. persicae* feeding on *Arabidopsis* revealed the induction of specific indolic GSs, such as 4-methoxyindol-3-ylmethyl glucosinolate, suggesting a crucial role in insect-host interactions even in the absence of myrosinase (Agerbirk et al., 2009). Another study in *Arabidopsis* showed that infestation by *M. persicae* and *B. brassicae* induces genes associated with indole GSs synthesis (Mewis et al., 2006; Kuśnierczyk et al., 2007), and *B. brassicae* infestation leads to GSs accumulation (Nouri-Ganbalani et al., 2018). However, contradictory results were observed in *B. juncea-fruticulosa* introgression lines infested by *L. erysimi*, with impacts on varying GS content (Palial et al., 2018). In *B. juncea*, transcripts related to biosynthetic pathways, including GSs, were induced in response to *A. craccivora*, whereas attenuated by *L. erysimi* infestation (Duhlian et al., 2020). In *B. rapa* infested by *L. erysimi*, the total GS content was enhanced, while those infested by *M. persicae* released lower levels (Blande et al., 2007). Slight stress by *B. brassicae* also enhanced leaf growth and increased GS emission in the bulb, the main storage organ of *B. rapa* (Sotelo et al., 2014). Considering the impact of herbivory on the GS-myrosinase defense system, sucking herbivores are known to trigger an increase in myrosinase enzyme activity or transcript levels (Siemens and Mitchell-Olds, 1998; Pontoppidan et al., 2005) or may have no effect (Travers-Martin and Müller, 2007). A separate study investigating the influence of *B. brassicae* infestation on the myrosinase-glucosinolate system in *B. napus* has reported induction of genes associated with this defense system (Pontoppidan et al., 2003). However, contradictory results have also been documented; for instance, in *Arabidopsis* infested by *B. brassicae*, a consistent decrease in myrosinase transcript levels was observed (Kuśnierczyk et al., 2007).

Similar to sucking herbivores, chewing herbivores significantly influence the regulation of GSs in plants. For example, generalist *S. exigua* and specialist *P. rapae* larvae, two recognized chewing herbivores, play a crucial role in shaping GS concentrations in different ecotypes of *Arabidopsis*. The impact is observed in both aliphatic and indole GSs, with a more pronounced effect on indole GS, consistent with patterns seen in herbivore-attacked plants (Mewis et al., 2006; Textor and Gershenzon, 2009; Gols et al., 2018). The feeding activity of *S. exigua* and *P. rapae* induces similar GS profiles after induction, demonstrating a consistent response to different chewing herbivores (Kos et al., 2012). In *B. oleracea*, *P. rapae* induces significant changes, leading to increased foliar concentrations of GSs compared to undamaged plants (Broekgaarden et al., 2007; Poelman et al., 2008). Caterpillar-induced slight stress on young *B. rapa* plants enhances bulb mass and results in a contrasting regulation of aliphatic and indolic GSs (Sotelo et al., 2014). Chewing herbivores’ impact on GS composition is evident in *Arabidopsis*, where *S. exigua* increases aliphatic GS content, and *P. rapae* slightly induces indole GSs. Additionally, *D. radicum* larval infestation upregulates indole GS synthesis genes in both low and high GS varieties of *B. rapa* (Sontowski et al., 2019). Recent studies on primary roots of *B. oleracea* reveal that leaf herbivores cause an increase in the expression of the indole GS biosynthesis gene *CYP81F4*, highlighting intricate regulatory mechanisms in plant defense against chewing herbivores (Karssemeijer et al., 2022). In summary, the intricate and varied responses in glucosinolate regulation underscore the specificity of plant-herbivore interactions and the adaptive strategies of *Brassica* plants to different feeding behaviors of insect herbivores (**Supplementary Table 1**).

### 3.5 Physical defenses in *Brassica* against insect herbivores

*Brassica* plants employ an array of physical defenses to shield themselves from herbivores and environmental challenges. These defenses encompass various components, including trichomes (Mathur et al., 2011; Hao et al., 2019), the cuticle (Khattab, 2007), the leaf surface (Eigenbrode and Espelie, 1995; Ahuja et al., 2010), and thorns or hairs (Traw, 2002) (**Figure 3**). It is worth noting that predictions suggest that both physical and chemical defense traits can be induced independently, without necessitating trade-offs. However, it is observed that the induction of physical traits may be comparatively weaker due to higher construction costs and time delays (Barton, 2016). These physical defense mechanisms, when combined with chemical defenses enhance the capacity to deter herbivores, form a comprehensive defense strategy for *Brassica* plants against herbivory and environmental stressors (Mostafa et al., 2022). Insect feeding patterns exert substantial influence on the physical defense mechanisms of *Brassica* plants, specifically impacting callose deposition, leaf thickness, and trichome density (Kos et al., 2012; Mathur et al., 2013b; Will et al., 2013; Rubil et al., 2022) (**Figure 3**). These responses typically manifest as alterations in trichome density, becoming noticeable within a timeframe of days to weeks (Dalin et al., 2008).

The impact of herbivore feeding on the physical defenses of *Brassica* plants has not been extensively studied. Only a limited number of investigations have been conducted, primarily focusing on chewing herbivores such as the larvae of *P. rapae* (Agren and Schemske, 1993; Traw, 2002; Traw and Dawson, 2002), *Trichoplusia ni* (Traw and Dawson, 2002), and *Spodoptera* species (Mathur et al., 2011). These studies have revealed that herbivore infestation significantly enhances the production of trichomes as a physical defense mechanism in *Brassica* plants against invading herbivores. In contrast, transcriptomic analysis of sucking herbivores feeding on *Brassica* plants revealed an induction in the gene expressions responsible for callose deposition [Callose synthase 1 (*CALS1*), vitamin C defective 2 (*VTC2*)], cell wall modifications [O-methyltransferase family 2 protein, vitamin C defective 2 (*VTC2*), and Xylogucan endotransglycosylase 6 (*XTH6*)], and trichome production [Glabrous 1 (*GL1*)] (Kempema et al., 2007; Kuśnierczyk et al., 2008; Broekgaarden et al., 2011) **Supplementary Table 1**.

The alteration of the cell wall, as observed in response to sucking herbivores, could discourage aphids by strengthening the barriers to probing (Thompson and Goggin, 2006). The host preference of *M. persicae* is impacted by *XTH* genes in *Arabidopsis* (Divol et al., 2007). Similarly, O-methyltransferase, found within the phenylpropanoid pathway, plays a role in the synthesis of lignin, a chemical compound known for imparting structural integrity to the cell wall (Whetten and Sederoff, 1995; Zhao et al., 2021). This function potentially serves as a defense mechanism against insects.

### 3.6 Other secondary metabolites in *Brassica*

In addition to extensively discussed compounds such as JA, SA, GSs, and physical defense mechanisms against sucking and chewing insect herbivores, it is crucial to acknowledge the significant contributions of other secondary metabolites. The secondary metabolites such as tannins, flavonoids, phenols, glycosides, terpenes, green leaf volatiles, phytoalexins, and camalexins are integral elements in the intricate defense strategies employed by *Brassica* plants against insect pests and pathogens (Simmonds, 2003; Ahuja et al., 2010; Cartea et al., 2010; Ibrahim et al., 2018). However, insect herbivory can alter their production and content in the plant. Chewing insect infestations, exemplified by flea beetles *Phyllotreta nemorum* and *P. brassicae* in *B. oleracea*, have been linked to an increase in polyphenols (Kovalikova et al., 2019). Additionally, *P. brassicae* caterpillar infestation in *B. oleracea* exhibited elevated levels of phenols, condensed tannins, and flavonoids, particularly in JA-treated plants (Ibrahim et al., 2018). The influence of *P. brassicae* caterpillar infestation on *B. nigra* resulted in significant alterations to sugars and phenolic compounds, with a specific impact on flavonol glucosides and hydroxycinnamic acid derivatives (Ponzio et al., 2017). Moreover, *P. brassicae* caterpillar feeding in *B. nigra* led to the accumulation of TCAs and phenylpropanoids while depleting amino acids (Papazian et al., 2019). Similarly, caterpillar infestation induces the emission of green-leaf volatiles and isothiocyanate in *B. rapa* (Verheggen et al., 2013).

Sap-sucking insect infestation also alters the secondary metabolite profile of *Brassica* plants. For example, aphid infestation in *Brassica* genotypes (*B. fruticulosa*, *B. juncea*, *B. rapa*) consistently led to a reduction in flavonols, total sugars, and free amino acids. Conversely, total phenols exhibited a reversed pattern, with a significant increase in phenol content in *Brassica* genotypes with minor exception for *B. fruticulosa* (Palial et al., 2018). Similarly, an induction in camalexin accumulation in *A. thaliana* has been recorded in response to *B. brassicae* infestation (Kuśnierczyk et al., 2008). In contrast, aphid *B. brassicae* feeding on *B. oleracea* resulted in a significant decrease in sugars, amino acids, and total soluble protein levels, accompanied by increased lipid peroxidation (malondialdehyde content) in infested leaves compared to healthy plants (Khattab, 2007). Notably, another study on *B. oleracea* found that aphid herbivory, induced by *B. brassicae* and the generalist *M. persicae*, did not affect the levels of flavonoids upon infestation (Khan et al., 2011). Additionally, numerous studies have reported an increase in the emission of volatile organic compounds (VOCs) from *Brassica* sp. plants upon insect herbivore infestation. These emissions differ in quality and quantity depending on the insect feeding guilds (Verheggen et al., 2013). For instance, *Brassica* sp. plants infested with aphids showed an elevated level of VOCs in blends, including terpenes (monoterpenes and sesquiterpenes), (E)-ß-farnesene, ß-pinene, and (E)-2-hexanol (Verheggen et al., 2013; Najar-Rodriguez et al., 2015). In summary, sap-sucking insect infestation induces complex changes in *Brassica* plants, affecting secondary metabolites and volatile organic compounds. This nuanced interaction highlights the diverse adaptive strategies employed by plants in response to insect herbivores.

Orchestrating signaling pathways, TFs interplay with the production of defensive compounds and structures in *Brassica* plants, playing a pivotal role in the intricate network of defense mechanisms against diverse insect feeding guilds. In *Brassica*, the transcription factors NPR1 and ETR1 are vital for enhancing total GS content in response to insect feeding (Mewis et al., 2005). Moreover, MYB transcription factors, specifically MYB28, MYB29, MYB34, and MYB122, play a pivotal role in elevating the expression of genes within the glucosinolate biosynthetic pathway, contributing to enhanced glucosinolate accumulation (Guo et al., 2013). The MYB/MYC model, involving MYB28, MYB29, MYC2, MYC3, and MYC4, influences aliphatic GS accumulation (Li et al., 2014). MYC2, a transcriptional activator in the MYC2-branch of the JA pathway, contributes to the wound-response and defense against insect herbivores (Verhage et al., 2011). Transcription factors, including MYB, NAC, WRKY, ERF (AtERF38), and MYC (bHLH), are pivotal in regulating secondary metabolites such as flavonoids and terpenoids, and contribute to the synthesis of lignin and cell wall thickening in plants (Owji et al., 2017; Wasternack and Strnad, 2019; Huang et al., 2023). R2R3 MYB, basic helix-loop-helix (bHLH), and WD40 proteins constitute major families regulating flavonoid and anthocyanin biosynthesis in *Brassica* (Chiu et al., 2010). Key transcription factors from the MYB and bHLH families, such as GLABRA1 (GL1), MYB5, MYB23, GLABRA3 (GL3), ENHANCER OF GLABRA3 (EGL3), and TRANSPARENT TESTA 8 (TT8), play a central role in regulating trichome development in *Brassica* (Chiu et al., 2010). Overexpressing NAC transcription factors NST1 and NST2 induces secondary wall thickening in *Brassica*, enhancing physical defense mechanisms (Yang et al., 2020). In conclusion, the interplay of transcription factors in *Brassica* plants, including NPR1, ETR1, MYBs, MYCs, and others, orchestrates a sophisticated defense network against diverse insect feeding guilds. Their regulatory roles span from glucosinolates and other secondary metabolite biosynthesis to different physical defense mechanisms, establishing a comprehensive and efficient response to biotic challenges.

## 4 Conclusion and future prospects

In summary, this review highlights the pivotal role of distinct defense mechanisms in *Brassica* plants when confronting chewing and sucking herbivores, involving JA-mediated pathways for the former and SA-mediated pathways for the latter. Additionally, we discuss how these pathways cross talk under herbivores attack. This specificity may enable the development of targeted pest management strategies, reducing reliance on environmentally harmful insecticides and promoting ecosystem sustainability. Categorizing defense responses into physical, chemical, and metabolic adjustments establishes a comprehensive framework for enhancing resilience to herbivores. The paper underscores the intricate interplay between herbivore feeding patterns and plant defense responses, providing valuable insights into the co-evolutionary dynamics between *Brassica* plants and insects.

Understanding the impact of insect herbivores’ diverse feeding patterns on plants involves a complex interplay of rapid and slow events at local and systemic levels. Recent findings by Ali et al., (2024), suggest that identifying these pathways enables the artificial induction of plant defense systems through mimicking the damage patterns caused by mechanical damage, thereby providing a controlled and sustainable approach. Investigating cross-talk between JA and SA pathways offers promise for developing a unified approach, allowing for specific adjustments based on insect feeding patterns, contributing to innovative and sustainable pest control methods. Tailoring plant defense strategies based on insights into insect feeding patterns can facilitate the development of resistant cultivars, optimizing plant resistance to prevalent herbivores in specific regions and improving crop success compared to non-resistant varieties. Analysing the relationship between insect herbivore feeding guilds and glucosinolate dynamics offers valuable genetic insights. This understanding can be leveraged to engineer resistant plant varieties through precise genetic modifications, such as gene knockouts or additions. These findings not only contribute to unravelling the plasticity of plant defenses against herbivores but also hold significance for the strategic management of *Brassica* in agroecosystems.

## Author contributions

JA: Conceptualization, Investigation, Software, Supervision, Visualization, Writing – original draft, Writing – review & editing. AT: Conceptualization, Investigation, Software, Supervision, Visualization, Writing – original draft, Writing – review & editing. TI: Writing – original draft, Writing – review & editing. SM: Writing – original draft, Writing – review & editing. MM: Funding acquisition, Writing – original draft, Writing – review & editing. AK: Funding acquisition, Writing – original draft, Writing – review & editing. RC: Writing – original draft, Writing – review & editing.

## References

[B1] Acevedo F. E.. (2015). Cues from chewing insects—the intersection of DAMPs, HAMPs, MAMPs and effectors. Curr. Opin. Plant Biol. 26, 80–86. doi: 10.1016/j.pbi.2015.05.029 26123394

[B2] Aftab T. Roychoudhury A. (2021). Crosstalk among plant growth regulators and signaling molecules during biotic and abiotic stresses: molecular responses and signaling pathways. Plant Cell Rep. 40, 2017–2019. doi: 10.1007/s00299-021-02791-5 34561762

[B3] Agerbirk N. De Vos M. Kim J. H. Jander G. (2009). Indole glucosinolate breakdown and its biological effects. Phytochem. Rev. 8, 101–120. doi: 10.1007/s11101-008-9098-0

[B4] Agerbirk N. Olsen C. E. (2012). Glucosinolate structures in evolution. Phytochemistry 77, 16–45. doi: 10.1016/j.phytochem.2012.02.005 22405332

[B5] Agrawal A. A. Kurashige N. S. (2003). A role for isothiocyanates in plant resistance against the specialist herbivore Pieris rapae. J. Chem. Ecol. 29, 1403–1415. doi: 10.1023/A:1024265420375 12918924

[B6] Agren J. Schemske D. W. (1993). The cost of defense against herbivores: an experimental study of trichome production in *Brassica rapa* . Am. Nat. 141, 338–350. doi: 10.1086/285477 19426086

[B7] Aharoni A. Giri A. P. Deuerlein S. Griepink F. de Kogel W.-J. Verstappen F. W. A.. (2003). Terpenoid metabolism in wild-type and transgenic Arabidopsis plants. Plant Cell 15, 2866–2884. doi: 10.1105/tpc.016253 14630967 PMC282818

[B8] Ahmed M. A. Cao H.-H. Jaleel W. Amir M. B. Ali M. Y. Smagghe G.. (2022). Oviposition preference and two-sex life table of *Plutella xylostella* and its association with defensive enzymes in three Brassicaceae crops. Crop Prot. 151, 105816. doi: 10.1016/j.cropro.2021.105816

[B9] Ahuja I. Rohloff J. Bones A. M. (2010). Defence mechanisms of Brassicaceae: Implications for plant-insect interactions and potential for integrated pest management. A review. Agron. Sustain. Dev. 30, 311–348. doi: 10.1051/agro/2009025

[B10] Ahuja I. Rohloff J. Bones A. M. (2011). Defence mechanisms of Brassicaceae: implications for plant-insect interactions and potential for integrated pest management. Sustain. Agric. 2, 623–670. doi: 10.1007/978-94-007-0394-0_28

[B11] Ali J. Mukarram M. Abbas A. Umar Md Fleischer P. Jr Mohamed H. I. (2024). Wound to survive: mechanical damage suppresses aphid performance on brassica. J. Plant Dis. Prot., 1–12. doi: 10.1007/s41348-024-00871-8

[B12] Aljbory Z. Chen M. (2018). Indirect plant defense against insect herbivores: a review. Insect Sci. 25, 2–23. doi: 10.1111/1744-7917.12436 28035791

[B13] Attia Z. Pogoda C. S. Reinert S. Kane N. C. Hulke B. S. (2021). Breeding for sustainable oilseed crop yield and quality in a changing climate. Theor. Appl. Genet. 134, 1817–1827. doi: 10.1007/s00122-021-03770-w 33496832

[B14] Badenes-Perez F. R. Gershenzon J. Heckel D. G. (2014). Insect attraction versus plant defense: young leaves high in glucosinolates stimulate oviposition by a specialist herbivore despite poor larval survival due to high saponin content. PloS One 9, e95766. doi: 10.1371/journal.pone.0095766 24752069 PMC3994119

[B15] Bakshi M. Oelmüller R. (2014). WRKY transcription factors: Jack of many trades in plants. Plant Signaling Behav. 9, e27700. doi: 10.4161/psb.27700 PMC4091213 24492469

[B16] Baldwin J. M. Paula-Moraes S. V. Mulvaney M. J. Meagher R. L. (2021). Occurrence of arthropod pests associated with *Brassica carinata* and impact of defoliation on yield. GCB Bioenergy 13, 570–581. doi: 10.1111/gcbb.12801

[B17] Bari R. Jones J. D. G. (2009). Role of plant hormones in plant defence responses. Plant Mol. Biol. 69, 473–488. doi: 10.1007/s11103-008-9435-0 19083153

[B18] Barth C. Jander G. (2006). Arabidopsis myrosinases TGG1 and TGG2 have redundant function in glucosinolate breakdown and insect defense. Plant J. 46, 549–562. doi: 10.1111/j.1365-313X.2006.02716.x 16640593

[B19] Barton K. E. (2016). Tougher and thornier: general patterns in the induction of physical defence traits. Funct. Ecol. 30, 181–187. doi: 10.1111/1365-2435.12495

[B20] Beekwilder J.. (2008). The impact of the absence of aliphatic glucosinolates on insect herbivory in Arabidopsis. PloS One 3, e2068. doi: 10.1371/journal.pone.0002068 18446225 PMC2323576

[B21] Blande J. D. Pickett J. A. Poppy G. M. (2007). A comparison of semiochemically mediated interactions involving specialist and generalist Brassica-feeding aphids and the braconid parasitoid *Diaeretiella rapae* . J. Chem. Ecol. 33, 767–779. doi: 10.1007/s10886-007-9264-7 17333371

[B22] Blenn B. Bandoly M. Küffner A. Otte T. Geiselhardt S. Fatouros N. E.. (2012). Insect egg deposition induces indirect defense and epicuticular wax changes in *Arabidopsis thaliana* . J. Chem. Ecol. 38, 882–892. doi: 10.1007/s10886-012-0132-8 22588570

[B23] Bodenhausen N. Reymond P. (2007). Signaling pathways controlling induced resistance to insect herbivores in Arabidopsis. Mol. Plant-Microbe Interact. 20, 1406–1420. doi: 10.1094/MPMI-20-11-1406 17977152

[B24] Bohinc T. Markovič D. Trdan S. (2014). Leaf epicuticular wax as a factor of antixenotic resistance of cabbage to cabbage flea beetles and cabbage stink bugs attack. Acta Agriculturae Scandinavica Section B—Soil Plant Sci. 64, 493–500. doi: 10.1080/09064710.2014.926978

[B25] Bonaventure G. (2012). Perception of insect feeding by plants. Plant Biol. 14, 872–880. doi: 10.1111/j.1438-8677.2012.00650.x 22957774

[B26] Bones A. M. Rossiter J. T. (1996). The myrosinase-glucosinolate system, its organisation and biochemistry. Physiologia plantarum 97, 194–208. doi: 10.1111/j.1399-3054.1996.tb00497.x

[B27] Broekgaarden C. Poelman E. H. Steenhuis G. Voorrips R. E. Dicke M. Vosman B. (2007). Genotypic variation in genome-wide transcription profiles induced by insect feeding: *Brassica oleracea–Pieris rapae* interactions. BMC Genomics 8, 1–13. doi: 10.1186/1471-2164-8-239 17640338 PMC1940009

[B28] Broekgaarden C. Voorrips R. E. Dicke M. Vosman B. (2011). Transcriptional responses of *Brassica nigra* to feeding by specialist insects of different feeding guilds. Insect Sci. 18, 259–272. doi: 10.1111/ins.2011.18.issue-3

[B29] Bruce T. J. A. (2014). Glucosinolates in oilseed rape: secondary metabolites that influence interactions with herbivores and their natural enemies. Ann. Appl. Biol. 164, 348–353. doi: 10.1111/aab.12128

[B30] Bruessow F. Gouhier-Darimont C. Buchala A. Metraux J.-P. Reymond P. (2010). Insect eggs suppress plant defence against chewing herbivores. Plant J. 62, 876–885. doi: 10.1111/tpj.2010.62.issue-5 20230509

[B31] Bruinsma M.. (2009). Jasmonic acid-induced volatiles of *Brassica oleracea* attract parasitoids: effects of time and dose, and comparison with induction by herbivores. J. Exp. Bot. 60, 2575–2587. doi: 10.1093/jxb/erp101 19451186 PMC2692006

[B32] Caarls L.. (2017). ‘Arabidopsis JASMONATE-INDUCED OXYGENASES down-regulate plant immunity by hydroxylation and inactivation of the hormone jasmonic acid’, Proc. Natl. Acad. Sci. U.S.A. 114, 6388–6393. doi: 10.1073/pnas.1701101114 PMC5474790 28559313

[B33] Cachapa J. C. Meyling N. V. Burow M. Hauser T. P. (2021). Induction and priming of plant defense by root-associated insect-pathogenic fungi. J. Chem. Ecol. 47, 112–122. doi: 10.1007/s10886-020-01234-x 33180275

[B34] Cao H. Bowling S. A. Gordon A. S. Dong X. (1994). Characterization of an Arabidopsis mutant that is nonresponsive to inducers of systemic acquired resistance. Plant Cell 6, 1583–1592. doi: 10.2307/3869945 12244227 PMC160545

[B35] Cardé R. T. Bell W. J. (1995). Chemical ecology of insects 2 (New York: Chapman & Hall), 433.

[B36] Cartea M. E. Francisco M. Soengas P. Velasco P. (2010). Phenolic compounds in Brassica vegetables. Molecules 16, 251–280. doi: 10.3390/molecules16010251 21193847 PMC6259264

[B37] Chauvin A. Caldelari D. Wolfender J.-L. Farmer E. E. (2013). Four 13-lipoxygenases contribute to rapid jasmonate synthesis in wounded *Arabidopsis thalian*a leaves: a role for lipoxygenase 6 in responses to long-distance wound signals. New Phytol. 197, 566–575. doi: 10.1111/nph.12029 23171345

[B38] Checker V. G. Kushwaha H. Kumari P. Yadav S. (2018). “Role of phytohormones in plant defense: signaling and cross talk,” in Molecular aspects of plant-pathogen interaction. Eds. Singh A. Singh I. (Springer, Singapore), 159–184. doi: 10.1007/978-981-10-7371-7_7

[B39] Chen M. S. (2008). Inducible direct plant defense against insect herbivores: A review. Insect Sci. 15, 101–114. doi: 10.1111/j.1744-7917.2008.00190.x 28035791

[B40] Chen L.. (2023). Comparative transcriptome analysis reveals a potential regulatory network for ogura cytoplasmic male sterility in cabbage (*Brassica oleracea* L.). Int. J. Mol. Sci. 24, 6703. doi: 10.3390/ijms24076703 37047676 PMC10094764

[B41] Chiu L.-W. Zhou X. Burke S. Wu X. Prior R. L. Li L. (2010). The purple cauliflower arises from activation of a MYB transcription factor. Plant Physiol. 154, 1470–1480. doi: 10.1104/pp.110.164160 20855520 PMC2971621

[B42] Cipollini D. Enright S. Traw M. B. Bergelson J. (2004). Salicylic acid inhibits jasmonic acid-induced resistance of *Arabidopsis thaliana* to *Spodoptera exigua* . Mol. Ecol. 13, 1643–1653. doi: 10.1111/j.1365-294X.2004.02161.x 15140107

[B43] Cole R. A. (1997). Comparison of feeding behaviour of two Brassica pests Brevicoryne brassicae and *Myzus persicae* on wild and cultivated Brassica species. Entomologia Experimentalis Applicata 85, 135–143. doi: 10.1046/j.1570-7458.1997.00243.x

[B44] Costarelli A.. (2020). Salicylic acid induced by herbivore feeding antagonizes jasmonic acid mediated plant defenses against insect attack. Plant Signaling Behav. 15, 1704517. doi: 10.1080/15592324.2019.1704517 PMC7012100 31852340

[B45] Dalin P. Ågren J. Björkman C. Huttunen P. Kärkkäinen K. (2008). “Leaf trichome formation and plant resistance to herbivory,” in Induced plant resistance to herbivory. Ed. Schaller A. (Springer, Netherland), 89–105. doi: 10.1007/978-1-4020-8182-8_4

[B46] De Geyter N. Gholami A. Goormachtig S. Goossens A. (2012). Transcriptional machineries in jasmonate-elicited plant secondary metabolism. Trends Plant Sci. 17, 349–359. doi: 10.1016/j.tplants.2012.03.001 22459758

[B47] Delaney T. P.. (1994). A central role of salicylic acid in plant disease resistance. Science 266, 1247–1250. doi: 10.1126/science.266.5188.1247 17810266

[B48] De Moraes C. M. Lewis W. J. Pare P. W. Alborn H. T. Tumiinson J. H. (1998). Herbivore-infested plants selectively attract parasitoids. Nature 393, 570–573. doi: 10.1038/31219

[B49] Dempsey D. A. Vlot C. Wildermuth M. C. Klessiga D. F. (2011). Salicylic acid biosynthesis and metabolism. Arabidopsis book/American Soc. Plant Biologists 9. doi: 10.1199/tab.0156 PMC3268552 22303280

[B50] Desveaux D.. (2004). A “Whirly” transcription factor is required for salicylic acid-dependent disease resistance in Arabidopsis. Dev. Cell 6, 229–240. doi: 10.1016/S1534-5807(04)00028-0 14960277

[B51] De Vos M.. (2005). Signal signature and transcriptome changes of Arabidopsis during pathogen and insect attack. Mol. Plant-Microbe Interact. 18, 923–937. doi: 10.1094/MPMI-18-0923 16167763

[B52] Dicke M. Beek T.A.V. Posthumus M. A. Dom N.B. Bokhoven H.V. De Groot Ae. (1990). Isolation and identification of volatile kairomone that affects acarine predator-prey interactions. J. Chem. Ecol. 16, 381–396. doi: 10.1007/BF01021772 24263497

[B53] Divol F. Vilaine F. Thibivilliers S. Kusiak C. Sauge M. H. Dinant S. (2007). Involvement of the xyloglucan endotransglycosylase/hydrolases encoded by celery XTH1 and Arabidopsis XTH33 in the phloem response to aphids. Plant Cell Environ. 30, 187–201. doi: 10.1111/j.1365-3040.2006.01618.x 17238910

[B54] Dombrecht B.. (2007). MYC2 differentially modulates diverse jasmonate-dependent functions in Arabidopsis. Plant Cell 19, 2225–2245. doi: 10.1105/tpc.106.048017 17616737 PMC1955694

[B55] Du Y. Poppy G. M. Powell W. Pickett J. A. Wadhams L. J. Woodcock C. M. (1998). Identification of semiochemicals released during aphid feeding that attract parasitoid *Aphidius ervi* . J. Chem. Ecol. 24, 1355–1368. doi: 10.1023/A:1021278816970

[B56] Duhlian L. Koramutla M. K. Subramanian S. Chamola R. Bhattacharya R. (2020). Comparative transcriptomics revealed differential regulation of defense related genes in *Brassica juncea* leading to successful and unsuccessful infestation by aphid species. Sci. Rep. 10, 10583. doi: 10.1038/s41598-020-66217-0 32601289 PMC7324606

[B57] Ederli L. Salerno G. Bianchet C. Rebora M. Piersanti S. Pasqualini S. (2020). *Eurydema oleracea* negatively affects defenses in Arabidopsis by inducing salicylic acid-mediated signaling pathway. Arthropod-plant Interact. 14, 139–148. doi: 10.1007/s11829-019-09728-6

[B58] Eigenbrode S. D. Espelie K. E. (1995). Effects of plant epicuticular lipids on insect herbivores. Annu. Rev. entomol. 40, 171–194. doi: 10.1146/annurev.en.40.010195.001131

[B59] Erb M. Meldau S. Howe G. A. (2012). Role of phytohormones in insect-specific plant reactions. Trends Plant Sci. 17, 250–259. doi: 10.1016/j.tplants.2012.01.003 22305233 PMC3346861

[B60] Erb M. Reymond P. (2019). Molecular interactions between plants and insect herbivores. Annu. Rev. Plant Biol. 70, 527–557. doi: 10.1146/annurev-arplant-050718-095910 30786233

[B61] Fatouros N. E. van Loon J. J. A. Hordijk K. A. Smid H. M. Dicke M. (2005). Herbivore-induced plant volatiles mediate in-flight host discrimination by parasitoids. J. Chem. Ecol. 31, 2033–2047. doi: 10.1007/s10886-005-6076-5 16132211

[B62] Fernández-Calvo P.. (2011). The Arabidopsis bHLH transcription factors MYC3 and MYC4 are targets of JAZ repressors and act additively with MYC2 in the activation of jasmonate responses. Plant Cell 23, 701–715. doi: 10.1105/tpc.110.080788 21335373 PMC3077776

[B63] Flint H. M. Salter S. S. Walters S. (1979). Caryophyllene: an attractant for the green lacewing. Environ. Entomol. 8, 1123–1125. doi: 10.1093/ee/8.6.1123

[B64] Fürstenberg-Hägg J. Zagrobelny M. Bak S. (2013). Plant defense against insect herbivores. Int. J. Mol. Sci. 14, 10242–10297. doi: 10.3390/ijms140510242 23681010 PMC3676838

[B65] Gatehouse J. A. (2002). Plant resistance towards insect herbivores: a dynamic interaction. New Phytol. 156, 145–169. doi: 10.1046/j.1469-8137.2002.00519.x 33873279

[B66] Gigolashvili T. Berger B. Mock H.-P. Müller C. Weisshaar B. Flügge U.-I. (2007a). The transcription factor HIG1/MYB51 regulates indolic glucosinolate biosynthesis in *Arabidopsis thaliana* . Plant J. 50, 886–901. doi: 10.1111/j.1365-313X.2007.03099.x 17461791

[B67] Gigolashvili T. Berger B. Flügge U.-I. (2009). Specific and coordinated control of indolic and aliphatic glucosinolate biosynthesis by R2R3-MYB transcription factors in *Arabidopsis thaliana* . Phytochem. Rev. 8, 3–13. doi: 10.1007/s11101-008-9112-6

[B68] Gigolashvili T. Engqvist M. Yatusevich R. Müller C. Flügge U.-I. (2008). HAG2/MYB76 and HAG3/MYB29 exert a specific and coordinated control on the regulation of aliphatic glucosinolate biosynthesis in *Arabidopsis thaliana* . New Phytol. 177, 627–642. doi: 10.1111/j.1469-8137.2007.02295.x 18042203

[B69] Gigolashvili T. Yatusevich R. Berger B. Müller C. Flügge U.-I. (2007b). The R2R3-MYB transcription factor HAG1/MYB28 is a regulator of methionine-derived glucosinolate biosynthesis in *Arabidopsis thaliana* . Plant J. 51, 247–261. doi: 10.1111/j.1365-313X.2007.03133.x 17521412

[B70] Glauser G. Grata E. Dubugnon L. Rudaz S. Farmer E. E. Wolfender J.-L. (2008). Spatial and temporal dynamics of jasmonate synthesis and accumulation in Arabidopsis in response to wounding. J. Biol. Chem. 283, 16400–16407. doi: 10.1074/jbc.M801760200 18400744

[B71] Gols R.. (2018). Seasonal and herbivore-induced dynamics of foliar glucosinolates in wild cabbage (*Brassica oleracea*). Chemoecology 28, 77–89. doi: 10.1007/s00049-018-0258-4 29904237 PMC5988764

[B72] Goossens J. Mertens J. Goossens A. (2017). Role and functioning of bHLH transcription factors in jasmonate signalling. J. Exp. Bot. 68, 1333–1347. doi: 10.1093/jxb/erw440 27927998

[B73] Guo R. Shen W. Qian H. Zhang M. Liu L. Wang Q. (2013). Jasmonic acid and glucose synergistically modulate the accumulation of glucosinolates in *Arabidopsis thaliana* . J. Exp. Bot. 64, 5707–5719. doi: 10.1093/jxb/ert348 24151308 PMC3871825

[B74] Guo Q.. (2018). ‘JAZ repressors of metabolic defense promote growth and reproductive fitness in Arabidopsis’, Proc. Natl. Acad. Sci. U.S.A. 115, E10768–E10777. doi: 10.1073/pnas.1811828115 PMC6233084 30348775

[B75] Hao Z.-P. Zhan H.-X. Wang Y.-L. Hou S.-M. (2019). How cabbage aphids *Brevicoryne brassicae* (L.) make a choice to feed on *Brassica napus* cultivars. Insects 10, 75. doi: 10.3390/insects10030075 30875981 PMC6468712

[B76] Heil M. (2008). Indirect defence via tritrophic interactions. New Phytol. 178, 41–61. doi: 10.1111/j.1469-8137.2007.02330.x 18086230

[B77] Hickman R.. (2019). Transcriptional dynamics of the salicylic acid response and its interplay with the jasmonic acid pathway. BioRxiv, 742742. doi: 10.1101/742742

[B78] Hopkins R. J. van Dam N. M. van Loon J. J. A. (2009). Role of glucosinolates in insect-plant relationships and multitrophic interactions. Annu. Rev. entomol. 54, 57–83. doi: 10.1146/annurev.ento.54.110807.090623 18811249

[B79] Hou S. Tsuda K. (2022). Salicylic acid and jasmonic acid crosstalk in plant immunity. Essays Biochem. 66, 647–656. doi: 10.1042/EBC20210090 35698792

[B80] Howe G. A. Jander G. (2008). Plant immunity to insect herbivores. Annu. Rev. Plant Biol. 59, 41–66. doi: 10.1146/annurev.arplant.59.032607.092825 18031220

[B81] Hu L.. (2010). Synthesis and antibacterial activity of C-12 pyrazolinyl spiro ketolides. Eur. J. medicinal Chem. 45, 5943–5949. doi: 10.1016/j.ejmech.2010.09.060 20970894

[B82] Huang Z. Xue Z. Zhao X. Wu C. Sun Y. Kou X. (2023). Transcription factors, potential regulatory targets in fruit defense responses to pathogens. Postharvest Biol. Technol. 206, 112589. doi: 10.1016/j.postharvbio.2023.112589

[B83] Ibrahim S. Mir G. M. Rouf A. War A. R. Hussain B. (2018). Herbivore and phytohormone induced defensive response in kale against cabbage butterfly, *Pieris brassicae* Linn. J. Asia-Pacific Entomol. 21, 367–373. doi: 10.1016/j.aspen.2018.01.018

[B84] Jagadeeswaran G.. (2007). Arabidopsis GH3-LIKE DEFENSE GENE 1 is required for accumulation of salicylic acid, activation of defense responses and resistance to *Pseudomonas syringae* . Plant J. 51, 234–246. doi: 10.1111/j.1365-313X.2007.03130.x 17521413

[B85] Jagodzik P. Tajdel-Zielinska M. Ciesla A. Marczak M. Ludwikow A. (2018). Mitogen-activated protein kinase cascades in plant hormone signaling. Front. Plant Sci. 9. doi: 10.3389/fpls.2018.01387 PMC6187979 30349547

[B86] Jamont M. Piva G. Fustec J. (2013). Sharing N resources in the early growth of rapeseed intercropped with faba bean: does N transfer matter? Plant Soil 371, 641–653. doi: 10.1007/s11104-013-1712-2

[B87] Jeschke V.. (2017). How glucosinolates affect generalist lepidopteran larvae: growth, development and glucosinolate metabolism. Front. Plant Sci. 8. doi: 10.3389/fpls.2017.01995 PMC5702293 29209354

[B88] Jeschke V.. (2021). So much for glucosinolates: A generalist does survive and develop on Brassicas, but at what cost? Plants 10, 962. doi: 10.3390/plants10050962 34066079 PMC8150600

[B89] Kappers I. F. Verstappen F. W.A. Luckerhoff L. L.P. Bouwmeester H. J. Dicke M. (2010). Genetic variation in jasmonic acid-and spider mite-induced plant volatile emission of cucumber accessions and attraction of the predator *Phytoseiulus persimilis* . J. Chem. Ecol. 36, 500–512. doi: 10.1007/s10886-010-9782-6 20383796 PMC2866305

[B90] Karban R. (2011). The ecology and evolution of induced resistance against herbivores. Funct. Ecol. 25, 339–347. doi: 10.1111/j.1365-2435.2010.01789.x

[B91] Kareiva P. (1999). Coevolutionary arms races: is victory possible? Proc. Natl. Acad. Sci. U.S.A. 96, 8–10. doi: 10.1073/pnas.96.1.8 9874761 PMC33539

[B92] Karssemeijer P. N. Winzen L. van Loon J. J. A. Dicke M. (2022). Leaf-chewing herbivores affect preference and performance of a specialist root herbivore. Oecologia 199, 243–255. doi: 10.1007/s00442-022-05132-9 35192063 PMC9226102

[B93] Katsir L. Schilmiller A. L. Staswick P. E. He S. Y. Howe G. A. (2008). COI1 is a critical component of a receptor for jasmonate and the bacterial virulence factor coronatine. Proc. Natl. Acad. Sci. U.S.A. 105, 7100–7105. doi: 10.1073/pnas.0802332105 18458331 PMC2383947

[B94] Kempema L. A. Cui X. Holzer F. M. Walling L. L. (2007). Arabidopsis transcriptome changes in response to phloem-feeding silverleaf whitefly nymphs. Similarities and distinctions in responses to aphids. Plant Physiol. 143, 849–865. doi: 10.1104/pp.106.090662 17189325 PMC1803730

[B95] Khan Z. R.. (1997). Intercropping increases parasitism of pests. Nature 388, 631–632. doi: 10.1038/41681

[B96] Khan M. A. M. Ulrichs C. Mewis I. (2011). Effect of water stress and aphid herbivory on flavonoids in broccoli (*Brassica oleracea* var. italica Plenck). J. Appl. Bot. Food Qual 84, 178–182.

[B97] Khattab H. (2007). The defense mechanism of cabbage plant against phloem-sucking aphid (*Brevicoryne brassicae* L.). Aust. J. Basic Appl. Sci. 1, 56–62.

[B98] Kim J. H. Lee B. W. Schroeder F. C. Jander G. (2008). Identification of indole glucosinolate breakdown products with antifeedant effects on *Myzus persicae* (green peach aphid). Plant J. 54, 1015–1026. doi: 10.1111/j.1365-313X.2008.03476.x 18346197

[B99] Kos M.. (2012). Herbivore-mediated effects of glucosinolates on different natural enemies of a specialist aphid. J. Chem. Ecol. 38, 100–115. doi: 10.1007/s10886-012-0065-2 22258357 PMC3268984

[B100] Kovalikova Z.. (2019). Changes in content of polyphenols and ascorbic acid in leaves of white cabbage after pest infestation. Molecules 24, 2622. doi: 10.3390/molecules24142622 31323864 PMC6680958

[B101] Kroes A. van Loon J. J. A. Dicke M. (2015). Density-dependent interference of aphids with caterpillar-induced defenses in Arabidopsis: involvement of phytohormones and transcription factors. Plant Cell Physiol. 56, 98–106. doi: 10.1093/pcp/pcu150 25339349

[B102] Kuśnierczyk A. Winge P. Jørstad T. S. Troczyńska J. Rossiter J. T. Bones A. M.. (2007). Transcriptional responses of *Arabidopsis thaliana* ecotypes with different glucosinolate profiles after attack by polyphagous *Myzus persicae* and oligophagous *Brevicoryne brassicae* . J. Exp. Bot. 58, 2537–2552. doi: 10.1093/jxb/erm043 17545220

[B103] Kuśnierczyk A. Winge P. Midelfart H. Armbruster W.S. Rossiter J. T. Bones A. M. (2008). Towards global understanding of plant defence against aphids–timing and dynamics of early Arabidopsis defence responses to cabbage aphid (*Brevicoryne brassicae*) attack. Plant Cell Environ. 31, 1097–1115. doi: 10.1111/j.1365-3040.2008.01823.x 18433442

[B104] Kundu P. Bera P. Mishra S. Vadassery J. (2023). ‘Regulatory role of phytohormones in the interaction of plants with insect herbivores’. Plant Hormones Crop Improvement, 41–64. doi: 10.1016/B978-0-323-91886-2.00003-3

[B105] Kunkel B. N. Brooks D. M. (2002). Cross talk between signaling pathways in pathogen defense. Curr. Opin. Plant Biol. 5, 325–331. doi: 10.1016/S1369-5266(02)00275-3 12179966

[B106] Laudert D. Schaller F. Weiler E. W. (2000). Transgenic *Nicotiana tabacum* and *Arabidopsis thaliana* plants overexpressing allene oxide synthase. Planta 211, 163–165. doi: 10.1007/s004250000316 10923718

[B107] Lebel E. Heifetz P. Thorne L. Uknes S. Ryals J. Ward E. (1998). Functional analysis of regulatory sequences controllingPR-1 gene expression in Arabidopsis. Plant J. 16, 223–233. doi: 10.1046/j.1365-313x.1998.00288.x 9839467

[B108] Li J. Brader G. Palva E. T. (2004). The WRKY70 transcription factor: a node of convergence for jasmonate-mediated and salicylate-mediated signals in plant defense. Plant Cell 16, 319–331. doi: 10.1105/tpc.016980 14742872 PMC341906

[B109] Li B.. (2014). Promoter-based integration in plant defense regulation. Plant Physiol. 166, 1803–1820. doi: 10.1104/pp.114.248716 25352272 PMC4256871

[B110] Little D. Gouhier-Darimont C. Bruessow F. Reymond P. (2007). Oviposition by pierid butterflies triggers defense responses in Arabidopsis. Plant Physiol. 143, 784–800. doi: 10.1104/pp.106.090837 17142483 PMC1803735

[B111] Louis J. Singh V. Shah J. (2012). *Arabidopsis thaliana*—aphid interaction. Arabidopsis book/American Soc. Plant Biologists 10. doi: 10.1199/tab.0159 PMC3365623 22666177

[B112] Mabry M. E.. (2021). The evolutionary history of wild, domesticated, and feral *Brassica oleracea* (Brassicaceae). Mol. Biol. Evol. 38, 4419–4434. doi: 10.1093/molbev/msab183 34157722 PMC8476135

[B113] Macaulay K. M.. (2017). The biochemical properties of the two *Arabidopsis thaliana* isochorismate synthases. Biochem. J. 474, 1579–1590. doi: 10.1042/BCJ20161069 28356402 PMC5408348

[B114] Mathur V. Ganta S. Raaijmakers C. E. Reddy A.S. Vet L. E. M. van Dam N. M. (2011). Temporal dynamics of herbivore-induced responses in *Brassica juncea* and their effect on generalist and specialist herbivores. Entomologia Experimentalis Applicata 139, 215–225. doi: 10.1111/j.1570-7458.2011.01122.x

[B115] Mathur V. Tytgat T. O. de Graaf R. M. Kalia V. Sankara Reddy A. Vet L. E.. (2013b). Dealing with double trouble: consequences of single and double herbivory in *Brassica juncea* . Chemoecology 23, 71–82. doi: 10.1007/s00049-012-0120-z

[B116] Mathur V. Tytgat T. O. G. Hordijk C. A. (2013c). An ecogenomic analysis of herbivore-induced plant volatiles in *Brassica juncea* . Mol. Ecol. 22, 6179–6196. doi: 10.1111/mec.12555 24219759

[B117] Mathur V. Wagenaar R. Caissard J. C. Reddy A. S. Vet L. E. Cortesero A. M.. (2013a). A novel indirect defence in Brassicaceae: structure and function of extrafloral nectaries in *Brassica juncea* . Plant Cell Environ. 36, 528–541. doi: 10.1111/j.1365-3040.2012.02593.x 22889298

[B118] Mauch-Mani B. Slusarenko A. J. (1996). Production of salicylic acid precursors is a major function of phenylalanine ammonia-lyase in the resistance of Arabidopsis to *Peronospora parasitica* . Plant Cell 8, 203–212. doi: 10.2307/3870265 12239383 PMC161092

[B119] Mewis I. Appel H. M. Hom A. Raina R. Schultz J. C. (2005). Major signaling pathways modulate Arabidopsis glucosinolate accumulation and response to both phloem-feeding and chewing insects. Plant Physiol. 138, 1149–1162. doi: 10.1104/pp.104.053389 15923339 PMC1150428

[B120] Mewis I. Tokuhis J. G. Schultz J. G. Appel H. M. Ulrichs C. Gershenzon J. (2006). Gene expression and glucosinolate accumulation in *Arabidopsis thaliana* in response to generalist and specialist herbivores of different feeding guilds and the role of defense signaling pathways. Phytochemistry 67, 2450–2462. doi: 10.1016/j.phytochem.2006.09.004 17049571

[B121] Millard P. S. Weber K. Kragelund B. B. Burow M. (2019). Specificity of MYB interactions relies on motifs in ordered and disordered contexts. Nucleic Acids Res. 47, 9592–9608. doi: 10.1093/nar/gkz691 31400117 PMC6765112

[B122] Mithofer A. Boland W. (2008). Recognition of herbivory-associated molecular patterns. Plant Physiol. 146, 825–831. doi: 10.1104/pp.107.113118 18316636 PMC2259064

[B123] Mitreiter S. Gigolashvili T. (2021). Regulation of glucosinolate biosynthesis. J. Exp. Bot. 72, 70–91. doi: 10.1093/jxb/eraa479 33313802

[B124] Moran P. J. Cheng Y. Cassell J. L. Thompson G. A. (2002). Gene expression profiling of *Arabidopsis thaliana* in compatible plant-aphid interactions. Arch. Insect Biochem. Physiol.: Published Collaboration Entomological Soc. America 51, 182–203. doi: 10.1002/arch.10064 12432519

[B125] Moran P. J. Thompson G. A. (2001). Molecular responses to aphid feeding in Arabidopsis in relation to plant defense pathways. Plant Physiol. 125, 1074–1085. doi: 10.1104/pp.125.2.1074 11161062 PMC64906

[B126] Moreno-Delafuente A. Garzo E. Fereres A. Viñuela E. Medina P. (2020). Effects of a salicylic acid analog on *Aphis gossypii* and its predator *Chrysoperla carnea* on melon plants. Agronomy 10, 1830. doi: 10.3390/agronomy10111830

[B127] Mostafa S. Wang Y. Zeng W. Jin B. (2022). Plant responses to herbivory, wounding, and infection. Int. J. Mol. Sci. 23, 7031. doi: 10.3390/ijms23137031 35806046 PMC9266417

[B128] Mumm R.. (2008). Formation of simple nitriles upon glucosinolate hydrolysis affects direct and indirect defense against the specialist herbivore, *Pieris rapae* . J. Chem. Ecol. 34, 1311–1321. doi: 10.1007/s10886-008-9534-z 18787901

[B129] Mur L. A. J. Kenton P. Atzorn R. Miersch O. Wasternack C. (2006). The outcomes of concentration-specific interactions between salicylate and jasmonate signaling include synergy, antagonism, and oxidative stress leading to cell death. Plant Physiol. 140, 249–262. doi: 10.1104/pp.105.072348 16377744 PMC1326048

[B130] Najar-Rodriguez A. J. Friedli M. Klaiber J. Dorn S. (2015). Aphid-deprivation from Brassica plants results in increased isothiocyanate release and parasitoid attraction. Chemoecology 25, 303–311. doi: 10.1007/s00049-015-0199-0

[B131] Nawrath C. Heck S. Parinthawong N. Métraux J. P. (2002). EDS5, an essential component of salicylic acid–dependent signaling for disease resistance in Arabidopsis, is a member of the MATE transporter family. Plant Cell 14, 275–286. doi: 10.1105/tpc.010376 11826312 PMC150564

[B132] Nawrath C. Métraux J.-P. (1999). Salicylic acid induction–deficient mutants of Arabidopsis express PR-2 and PR-5 and accumulate high levels of camalexin after pathogen inoculation. Plant Cell 11, 1393–1404. doi: 10.1105/tpc.11.8.1393 10449575 PMC144293

[B133] Newton E. L. Bullock J. M. Hodgson D. J. (2009). Glucosinolate polymorphism in wild cabbage (*Brassica oleracea*) influences the structure of herbivore communities. Oecologia 160, 63–76. doi: 10.1007/s00442-009-1281-5 19214588

[B134] Nguyen D. Rieu I. Mariani C. van Dam N. M. (2016). How plants handle multiple stresses: hormonal interactions underlying responses to abiotic stress and insect herbivory. Plant Mol. Biol. 91, 727–740. doi: 10.1007/s11103-016-0481-8 27095445 PMC4932144

[B135] Nobuta K. Okrent R. A. Stoutemyer M. Rodibaugh N. Kempema L. Wildermuth M. C.. (2007). The GH3 acyl adenylase family member PBS3 regulates salicylic acid-dependent defense responses in Arabidopsis. Plant Physiol. 144, 1144–1156. doi: 10.1104/pp.107.097691 17468220 PMC1914169

[B136] Nomoto M. Skelly M. J. Itaya T. Mori T. Suzuki T. Matsushita T.. (2021). Suppression of MYC transcription activators by the immune cofactor NPR1 fine-tunes plant immune responses. Cell Rep. 37. doi: 10.1016/j.celrep.2021.110125 34910911

[B137] Nouri-Ganbalani G. Borzoui E. Shahnavazi M. (2018). Induction of resistance against *Plutella xylostella* (L.)(Lep.: Plutellidae) by jasmonic acid and mealy cabbage aphid feeding in *Brassica napus* L. Front. Physiol. 9. doi: 10.3389/fphys.2018.00859 PMC6052903 30050454

[B138] Nürnberger T. Scheel D. (2001). Signal transmission in the plant immune response. Trends Plant Sci. 6, 372–379. doi: 10.1016/S1360-1385(01)02019-2 11495791

[B139] Onkokesung N. Reichelt M. van Doorn A. Schuurink R. C. Dicke M. (2016). Differential costs of two distinct resistance mechanisms induced by different herbivore species in Arabidopsis. Plant Physiol. 170, 891–906. doi: 10.1104/pp.15.01780 26603653 PMC4734589

[B140] Orlovskis Z. Reymond P. (2020). Pieris brassicae eggs trigger interplant systemic acquired resistance against a foliar pathogen in Arabidopsis. New Phytol. 228, 1652–1661. doi: 10.1111/nph.16788 32619278

[B141] Owji H. Hajiebrahimi A. Seradj H. Hemmati S. (2017). Identification and functional prediction of stress responsive AP2/ERF transcription factors in *Brassica napus* by genome-wide analysis. Comput. Biol. Chem. 71, 32–56. doi: 10.1016/j.compbiolchem.2017.09.004 28961511

[B142] Ozawa R. Shiojiri K. Sabelis M. W. Takabayashi J. (2008). Maize plants sprayed with either jasmonic acid or its precursor, methyl linolenate, attract armyworm parasitoids, but the composition of attractants differs. Entomologia Experimentalis Applicata 129, 189–199. doi: 10.1111/j.1570-7458.2008.00767.x

[B143] Palial S. Kumar S. Sharma S. (2018). Biochemical changes in the *Brassica juncea*-fruticulosa introgression lines after *Lipaphis erysimi* (Kaltenbach) infestation. Phytoparasitica 46, 499–509. doi: 10.1007/s12600-018-0686-2

[B144] Papazian S. Girdwood T. Wessels B. A. Poelman E. H. Dicke M. Moritz T.. (2019). Leaf metabolic signatures induced by real and simulated herbivory in black mustard (*Brassica nigra*). Metabolomics 15, 1–16. doi: 10.1007/s11306-019-1592-4 PMC6765471 31563978

[B145] Patra B. Pattanaik S. Schluttenhofer C. Yuan L. (2018). A network of jasmonate-responsive bHLH factors modulate monoterpenoid indole alkaloid biosynthesis in *Catharanthus roseus* . New Phytol. 217, 1566–1581. doi: 10.1111/nph.14910 29178476

[B146] Pieterse C. M. J. Leon-Reyes A. van der Ent S. Van Wees S. C. (2009). Networking by small-molecule hormones in plant immunity. Nat. Chem. Biol. 5, 308–316. doi: 10.1038/nchembio.164 19377457

[B147] Pieterse C. M. J. Van Loon L. C. (2004). NPR1: the spider in the web of induced resistance signaling pathways. Curr. Opin. Plant Biol. 7, 456–464. doi: 10.1016/j.pbi.2004.05.006 15231270

[B148] Poelman E. H. Broekgaarden C. Van Loon J. J. Dicke M. (2008). Early season herbivore differentially affects plant defence responses to subsequently colonizing herbivores and their abundance in the field. Mol. Ecol. 17, 3352–3365. doi: 10.1111/j.1365-294X.2008.03838.x 18565114

[B149] Pontoppidan B. Hopkins R. Rask L. Meijer J. (2003). Infestation by cabbage aphid (*Brevicoryne brassicae*) on oilseed rape (*Brassica napus*) causes a long lasting induction of the myrosinase system. Entomologia Experimentalis Applicata 109, 55–62. doi: 10.1046/j.1570-7458.2003.00088.x

[B150] Pontoppidan B. Hopkins R. Rask L. Meijer J. (2005). Differential wound induction of the myrosinase system in oilseed rape (*Brassica napus*): contrasting insect damage with mechanical damage. Plant Sci. 168, 715–722. doi: 10.1016/j.plantsci.2004.10.003

[B151] Ponzio C. Papazian S. Albrectsen B. R. Dicke M. Gols R. (2017). Dual herbivore attack and herbivore density affect metabolic profiles of *Brassica nigra* leaves. Plant Cell Environ. 40, 1356–1367. doi: 10.1111/pce.12926 28155236

[B152] Poveda J. Francisco M. Cartea M. E. Velasco P. (2020). Development of transgenic Brassica crops against biotic stresses caused by pathogens and arthropod pests. Plants 9, 1664. doi: 10.3390/plants9121664 33261092 PMC7761317

[B153] Puente M. Magori K. Kennedy G. G. Gould F. (2008). Impact of herbivore-induced plant volatiles on parasitoid foraging success: a spatial simulation of the *Cotesia rubecula*, *Pieris rapae*, and *Brassica oleracea* system. J. Chem. Ecol. 34, 959–970. doi: 10.1007/s10886-008-9472-9 18438615

[B154] Qi T. Song S. Ren Q. Wu D. Huang H. Chen Y.. (2011). The Jasmonate-ZIM-domain proteins interact with the WD-Repeat/bHLH/MYB complexes to regulate Jasmonate-mediated anthocyanin accumulation and trichome initiation in *Arabidopsis thaliana* . Plant Cell 23, 1795–1814. doi: 10.1105/tpc.111.083261 21551388 PMC3123955

[B155] Raffaele S. Rivas S. Roby D. (2006). An essential role for salicylic acid in AtMYB30-mediated control of the hypersensitive cell death program in Arabidopsis. FEBS Lett. 580, 3498–3504. doi: 10.1016/j.febslet.2006.05.027 16730712

[B156] Rasmann S. Köllner T. G. Degenhardt J. Hiltpold I. Toepfer S. Kuhlmann U.. (2005). Recruitment of entomopathogenic nematodes by insect-damaged maize roots. Nature 434, 732–737. doi: 10.1038/nature03451 15815622

[B157] Ratzka A. Vogel H. Kliebenstein D. J. Mitchell-Olds T. Kroymann J. (2002). Disarming the mustard oil bomb. Proc. Natl. Acad. Sci. U.S.A. 99, 11223–11228. doi: 10.1073/pnas.172112899 12161563 PMC123237

[B158] Rejeb I.B. Pastor V. Mauch-Mani B. (2014). Plant responses to simultaneous biotic and abiotic stress: molecular mechanisms. Plants 3, 458–475. doi: 10.3390/plants3040458 27135514 PMC4844285

[B159] Rekhter D. Lüdke D. Ding Y. Feussner K. Zienkiewicz K. Lipka V.. (2019). Isochorismate-derived biosynthesis of the plant stress hormone salicylic acid. Science 365, 498–502. doi: 10.1126/science.aaw1720 31371615

[B160] Ruan J. Zhou Y. Zhou M. Yan J. Khurshid M. Weng W.. (2019). Jasmonic acid signaling pathway in plants. Int. J. Mol. Sci. 20, 2479. doi: 10.3390/ijms20102479 31137463 PMC6566436

[B161] Rubil N. Kalachova T. Hauser T. P. Burketová L. (2022). Specialist aphid feeding causes local activation of salicylic and jasmonic acid signaling in Arabidopsis veins. Mol. Plant-Microbe Interact. 35, 119–124. doi: 10.1094/MPMI-08-21-0203-SC 34669427

[B162] Sato Y. Tezuka A. Kashima M. Deguchi A. Shimizu-Inatsugi R. Yamazaki M.. (2019). Transcriptional variation in glucosinolate biosynthetic genes and inducible responses to aphid herbivory on field-grown *Arabidopsis thaliana* . Front. Genet. 10. doi: 10.3389/fgene.2019.00787 PMC6749069 31572432

[B163] Schmiesing A. Emonet A. Gouhier-Darimont C. Reymond P. (2016). Arabidopsis MYC transcription factors are the target of hormonal salicylic acid/jasmonic acid cross talk in response to *Pieris brassicae* egg extract. Plant Physiol. 170, 2432–2443. doi: 10.1104/pp.16.00031 26884488 PMC4825139

[B164] Schoonhoven L. M. Van Loon J. J. Dicke M. (2005). Insect-plant biology (Oxford, UK: Oxford University Press). doi: 10.1093/oso/9780198525943.001.0001

[B165] Schweizer F. Fernández-Calvo P. Zander M. Diez-Diaz M. Fonseca S. Glauser G.. (2013). Arabidopsis basic helix-loop-helix transcription factors MYC2, MYC3, and MYC4 regulate glucosinolate biosynthesis, insect performance, and feeding behavior. Plant Cell 25, 3117–3132. doi: 10.1105/tpc.113.115139 23943862 PMC3784603

[B166] Seo S. Okamoto M. Seto H. Ishizuka K. Sano H. Ohashi Y. (1995). Tobacco MAP kinase: a possible mediator in wound signal transduction pathways. Science 270, 1988–1992. doi: 10.1126/science.270.5244.1988 8533090

[B167] Seo M.-S. Kim J. S. (2017). Understanding of MYB transcription factors involved in glucosinolate biosynthesis in Brassicaceae. Molecules 22, 1549. doi: 10.3390/molecules22091549 28906468 PMC6151624

[B168] Shah J. Tsui F. Klessig D. F. (1997). Characterization of a salicylic acid-insensitive mutant (sai1) of *Arabidopsis thaliana*, identified in a selective screen utilizing the SA-inducible expression of the tms2 gene. Mol. Plant-Microbe Interact. 10, 69–78. doi: 10.1094/MPMI.1997.10.1.69 9002272

[B169] Sheard L. B. Tan X. Mao H. Withers J. Ben-Nissan G. Hinds T. R.. (2010). Jasmonate perception by inositol-phosphate-potentiated COI1–JAZ co-receptor. Nature 468, 400–405. doi: 10.1038/nature09430 20927106 PMC2988090

[B170] Siemens D. H. Mitchell-Olds T. (1998). Evolution of pest-induced defenses in Brassica plants: tests of theory. Ecology 79, 632–646. doi: 10.1890/0012-9658(1998)079[0632:EOPIDI]2.0.CO;2

[B171] Silva G. A. Pereira R. M. Rodrigues-Silva N. Souza T. C. Ferreira D. O. Queiroz E. A.. (2017). Wax removal and diamondback moth performance in collards cultivars. Neotropical Entomol. 46, 571–577. doi: 10.1007/s13744-017-0493-3 28478539

[B172] Simmonds M. S. J. (2003). Flavonoid–insect interactions: recent advances in our knowledge. Phytochemistry 64, 21–30. doi: 10.1016/S0031-9422(03)00293-0 12946403

[B173] Smith C. M. Boyko E. V. (2007). The molecular bases of plant resistance and defense responses to aphid feeding: current status. Entomologia experimentalis applicata 122, 1–16. doi: 10.1111/j.1570-7458.2006.00503.x

[B174] Sontowski R. Gorringe N. J. Pencs S. Schedl A. Touw A. J. Van Dam N. M. (2019). Same difference? Low and high glucosinolate *Brassica rapa* varieties show similar responses upon feeding by two specialist root herbivores. Front. Plant Sci. 10. doi: 10.3389/fpls.2019.01451 PMC6865846 31798608

[B175] Sotelo P. Pérez E. Najar-Rodriguez A. Walter A. Dorn S. (2014). Brassica plant responses to mild herbivore stress elicited by two specialist insects from different feeding guilds. J. Chem. Ecol. 40, 136–149. doi: 10.1007/s10886-014-0386-4 24500734

[B176] Spoel S. H. Dong X. (2008). Making sense of hormone crosstalk during plant immune responses. Cell Host Microbe 3, 348–351. doi: 10.1016/j.chom.2008.05.009 18541211

[B177] Spoel S. H. Koornneef A. Claessens S. M. Korzelius J. P. Van Pelt J. A. Mueller M. J.. (2003). NPR1 modulates cross-talk between salicylate-and jasmonate-dependent defense pathways through a novel function in the cytosol. Plant Cell 15, 760–770. doi: 10.1105/tpc.009159 12615947 PMC150028

[B178] Spoel S. H. Mou Z. Tada Y. Spivey N. W. Genschik P. Dong X. (2009). Proteasome-mediated turnover of the transcription coactivator NPR1 plays dual roles in regulating plant immunity. Cell 137, 860–872. doi: 10.1016/j.cell.2009.03.038 19490895 PMC2704463

[B179] Spoel S. H. Johnson J. S. Dong X. (2007). Regulation of tradeoffs between plant defenses against pathogens with different lifestyles. Proc. Natl. Acad. Sci. U.S.A. 104, 18842–18847. doi: 10.1073/pnas.0708139104 17998535 PMC2141864

[B180] Stahl E. Hilfiker O. Reymond P. (2018). Plant–arthropod interactions: who is the winner? Plant J. 93, 703–728. doi: 10.1111/tpj.13773 29160609

[B181] Staswick P. E. Tiryaki I. (2004). The oxylipin signal jasmonic acid is activated by an enzyme that conjugates it to isoleucine in Arabidopsis. Plant Cell 16, 2117–2127. doi: 10.1105/tpc.104.023549 15258265 PMC519202

[B182] Strawn M. A. Marr S. K. Inoue K. Inada N. Zubieta C. Wildermuth M. C. (2007). Arabidopsis isochorismate synthase functional in pathogen-induced salicylate biosynthesis exhibits properties consistent with a role in diverse stress responses. J. Biol. Chem. 282, 5919–5933. doi: 10.1074/jbc.M605193200 17190832

[B183] Stroud E. A. Jayaraman J. Templeton M. D. Rikkerink E. H. (2022). Comparison of the pathway structures influencing the temporal response of salicylate and jasmonate defence hormones in *Arabidopsis thaliana* . Front. Plant Sci. 13. doi: 10.3389/fpls.2022.952301 PMC9504473 36160984

[B184] Suza W. P. Staswick P. E. (2008). The role of JAR1 in jasmonoyl-L-isoleucine production during Arabidopsis wound response. Planta 227, 1221–1232. doi: 10.1007/s00425-008-0694-4 18247047

[B185] Textor S. Gershenzon J. (2009). Herbivore induction of the glucosinolate–myrosinase defense system: major trends, biochemical bases and ecological significance. Phytochem. Rev. 8, 149–170. doi: 10.1007/s11101-008-9117-1

[B186] Thaler J. S. Humphrey P. T. Whiteman N. K. (2012). Evolution of jasmonate and salicylate signal crosstalk. Trends Plant Sci. 17, 260–270. doi: 10.1016/j.tplants.2012.02.010 22498450

[B187] Tholl D. Lee S. (2011). Terpene specialized metabolism in *Arabidopsis thaliana* . Arabidopsis Book/American Soc. Plant Biologists 9. doi: 10.1199/tab.0143 PMC3268506 22303268

[B188] Thompson G. A. Goggin F. L. (2006). Transcriptomics and functional genomics of plant defence induction by phloem-feeding insects. J. Exp. Bot. 57, 755–766. doi: 10.1093/jxb/erj135 16495409

[B189] Titarenko E. Rojo E. Leon J. Sanchez-Serrano J. J. (1997). Jasmonic acid-dependent and-independent signaling pathways control wound-induced gene activation in *Arabidopsis thaliana* . Plant Physiol. 115, 817–826. doi: 10.1104/pp.115.2.817 9342878 PMC158541

[B190] Torrens-Spence M. P. Bobokalonova A. Carballo V. Glinkerman C. M. Pluskal T. Shen A.. (2019). PBS3 and EPS1 complete salicylic acid biosynthesis from isochorismate in Arabidopsis. Mol. Plant 12, 1577–1586. doi: 10.1016/j.molp.2019.11.005 31760159

[B191] Touw A. J. Verdecia Mogena A. Maedicke A. Sontowski R. Van Dam N. M. Tsunoda T. (2020). Both biosynthesis and transport are involved in glucosinolate accumulation during root-herbivory in *Brassica rapa* . Front. Plant Sci. 10. doi: 10.3389/fpls.2019.01653 PMC6970201 31998341

[B192] Travers-Martin N. Müller C. (2007). Specificity of induction responses in *Sinapis alba* L. and their effects on a specialist herbivore. J. Chem. Ecol. 33, 1582–1597. doi: 10.1007/s10886-007-9322-1 17587140

[B193] Traw M. B. (2002). Is induction response negatively correlated with constitutive resistance in black mustard? Evolution 56, 2196–2205. doi: 10.1111/j.0014-3820.2002.tb00144.x 12487350

[B194] Traw B. M. Dawson T. E. (2002). Differential induction of trichomes by three herbivores of black mustard. Oecologia 131, 526–532. doi: 10.1007/s00442-002-0924-6 28547547

[B195] Tsuda K. Sato M. Stoddard T. Glazebrook J. Katagiri F. (2009). Network properties of robust immunity in plants. PloS Genet. 5, e1000772. doi: 10.1371/journal.pgen.1000772 20011122 PMC2782137

[B196] Uefune M. Kugimiya S. Sano K. Takabayashi J. (2012). Herbivore-induced plant volatiles enhance the ability of parasitic wasps to find hosts on a plant. J. Appl. Entomol. 136, 133–138. doi: 10.1111/j.1439-0418.2011.01621.x

[B197] Uemura T. Arimura G.-I. (2019). Current opinions about herbivore-associated molecular patterns and plant intracellular signaling. Plant Signaling Behav. 14, e1633887. doi: 10.1080/15592324.2019.1633887 PMC6768233 31230525

[B198] van Dam N. M. Raaijmakers C. E. (2006). Local and systemic induced responses to cabbage root fly larvae (*Delia radicum*) in Brassica nigra and B. oleracea. Chemoecology 16, 17–24. doi: 10.1007/s00049-005-0323-7

[B199] van Loon J. J. A. de Boer J. G. Dicke M. (2000). Parasitoid-plant mutualism: parasitoid attack of herbivore increases plant reproduction. Entomologia experimentalis applicata 97, 219–227. doi: 10.1046/j.1570-7458.2000.00733.x

[B200] van Poecke R. M. P. (2007). Arabidopsis-insect interactions. Arabidopsis Book/American Soc. Plant Biologists 5. doi: 10.1199/tab.0107 PMC3243410 22303231

[B201] van Poecke R. M. P. Dicke M. (2002). Induced parasitoid attraction by *Arabidopsis thaliana* : involvement of the octadecanoid and the salicylic acid pathway. J. Exp. Bot. 53, 1793–1799. doi: 10.1093/jxb/erf022 12147729

[B202] van Poecke R. M. P. Posthumus M. A. Dicke M. (2001). Herbivore-induced volatile production by *Arabidopsis thaliana* leads to attraction of the parasitoid *Cotesia rubecula*: chemical, behavioral, and gene-expression analysis. J. Chem. Ecol. 27, 1911–1928. doi: 10.1023/A:1012213116515 11710601

[B203] Vega-Álvarez C. Francisco M. Cartea M. E. Fernández J. C. Soengas P. (2023). The growth-immunity tradeoff in *Brassica oleracea*-*Xanthomonas campestris* pv. campestris pathosystem. Plant Cell Environ. 46, 2985–2997. doi: 10.1111/pce.14454 36180381

[B204] Verhage A. Vlaardingerbroek I. Raaijmakers C. Van Dam N. Dicke M. Van Wees S. C.. (2011). Rewiring of the jasmonate signaling pathway in Arabidopsis during insect herbivory. Front. Plant Sci. 2. doi: 10.3389/fpls.2011.00047 PMC3355780 22645537

[B205] Verheggen F. J. Haubruge E. De Moraes C. M. Mescher M. C. (2013). Aphid responses to volatile cues from turnip plants (*Brassica rapa*) infested with phloem-feeding and chewing herbivores. Arthropod-Plant Interact. 7, 567–577. doi: 10.1007/s11829-013-9272-1

[B206] Verma V. Ravindran P. Kumar P. P. (2016). Plant hormone-mediated regulation of stress responses. BMC Plant Biol. 16, 1–10. doi: 10.1186/s12870-016-0771-y 27079791 PMC4831116

[B207] Vos I. A. Moritz L. Pieterse C. M. Van Wees S. C. (2013). Onset of herbivore-induced resistance in systemic tissue primed for jasmonate-dependent defenses is activated by abscisic acid. Front. Plant Sci. 4. doi: 10.3389/fpls.2013.00539 PMC3874679 24416038

[B208] Vos I. A. Verhage A. Schuurink R. C. Pieterse C. M. Van Wees S. C. (2015). Impact of hormonal crosstalk on plant resistance and fitness under multi-attacker conditions. Front. Plant Sci. 6. doi: 10.3389/fpls.2015.00639 PMC4538242 26347758

[B209] Walling L. L. (2000). The myriad plant responses to herbivores. J. Plant Growth Regul. 19, 195–216. doi: 10.1007/s003440000026 11038228

[B210] Walling L. L. (2008). Avoiding effective defenses: strategies employed by phloem-feeding insects. Plant Physiol. 146, 859–866. doi: 10.1104/pp.107.113142 18316641 PMC2259051

[B211] War A. R. Kabir M. A. Mujiono K. Hojo Y. Shinya T. Tani A.. (2012). Mechanisms of plant defense against insect herbivores. Plant Signaling Behav. 7. doi: 10.4161/psb.21663 PMC3493419 22895106

[B212] War A. R. Paulraj M. G. Ahmad T. Buhroo A. A. Hussain B. Ignacimuthu S.. (2018). Plant defence against herbivory and insect adaptations. AoB Plants 10, ply037. doi: 10.1093/aobpla/ply037

[B213] Wari D. Kabir M. A. Mujiono K. Hojo Y. Shinya T. Tani A.. (2019). Honeydew-associated microbes elicit defense responses against brown planthopper in rice. J. Exp. Bot. 70, 1683–1696. doi: 10.1093/jxb/erz041 30715410 PMC6411376

[B214] Warwick S. I. (2011). “Brassicaceae in agriculture,” in Genetics and Genomics of the Brassicaceae. Eds. Schmidt R. Bancroft I. (Springer, New York), 33–65. doi: 10.1007/978-1-4419-7118-0_2

[B215] Wasternack C. Hause B. (2013). Jasmonates : Biosynthesis, perception, signal transduction and action in plant stress response, growth and development. An update to the 2007 review in Annals of Botany. Ann. Bot. 111, 1021–1058. doi: 10.1093/aob/mct067 23558912 PMC3662512

[B216] Wasternack C. Strnad M. (2019). Jasmonates are signals in the biosynthesis of secondary metabolites—Pathways, transcription factors and applied aspects—A brief review. New Biotechnol. 48, 1–11. doi: 10.1016/j.nbt.2017.09.007 29017819

[B217] Weech M. H. Chapleau M. Pan L. Ide C. Bede J. C. (2008). Caterpillar saliva interferes with induced *Arabidopsis thaliana* defence responses via the systemic acquired resistance pathway. J. Exp. Bot. 59, 2437–2448. doi: 10.1093/jxb/ern108 18487634 PMC2423655

[B218] Weigel R. R. Bäuscher C. Pfitzner A. J. Pfitzner U. M. (2001). NIMIN-1, NIMIN-2 and NIMIN-3, members of a novel family of proteins from Arabidopsis that interact with NPR1/NIM1, a key regulator of systemic acquired resistance in plants. Plant Mol. Biol. 46, 143–160. doi: 10.1023/A:1010652620115 11442055

[B219] Weigel R. R. Pfitzner U. M. Gatz C. (2005). Interaction of NIMIN1 with NPR1 modulates PR gene expression in Arabidopsis. Plant Cell 17, 1279–1291. doi: 10.1105/tpc.104.027441 15749762 PMC1088002

[B220] Whetten R. Sederoff R. (1995). Lignin biosynthesis. Plant Cell 7, 1001. doi: 10.1105/tpc.7.7.1001 12242395 PMC160901

[B221] Wildermuth M. C. Dewdney J. Wu G. Ausubel F. M. (2001). Isochorismate synthase is required to synthesize salicylic acid for plant defence. Nature 414, 562–565. doi: 10.1038/35107108 11734859

[B222] Will T. Furch A. C. U. Zimmermann M. R. (2013). How phloem-feeding insects face the challenge of phloem-located defenses. Front. Plant Sci. 4. doi: 10.3389/fpls.2013.00336 PMC3756233 24009620

[B223] Wittstock U. Agerbirk N. Stauber E. J. Olsen C. E. Hippler M. Mitchell-Olds T.. (2004). Successful herbivore attack due to metabolic diversion of a plant chemical defense. Proc. Natl. Acad. Sci. U.S.A. 101, 4859–4864. doi: 10.1073/pnas.0308007101 15051878 PMC387339

[B224] Wittstock U. Kurzbach E. Herfurth A. M. Stauber E. J. (2016). Glucosinolate breakdown’. Adv. botanical Res. 80, 125–169. doi: 10.1016/bs.abr.2016.06.006

[B225] Xiao Y. Wang Q. Erb M. Turlings T. C. Ge L. Hu L.. (2012). Specific herbivore-induced volatiles defend plants and determine insect community composition in the field. Ecol. Lett. 15, 1130–1139. doi: 10.1111/j.1461-0248.2012.01835.x 22804824

[B226] Yan J. Li H. Li S. Yao R. Deng H. Xie Q.. (2013). The Arabidopsis F-box protein CORONATINE INSENSITIVE1 is stabilized by SCFCOI1 and degraded via the 26S proteasome pathway. Plant Cell 25, 486–498. doi: 10.1105/tpc.112.105486 23386265 PMC3608773

[B227] Yang S. Wei J. Yang S. Kuang R. (2011). Current Status and Future Trends of Augmentative Release of *Aphidius gifuensis* for Control of *Myzus persicae* in China’s Yunnan Province. J. Entomological Res. Soc. 13, 87–99.

[B228] Yang J. Chen H. Yang C. Ding Q. Zhao T. Wang D. (2020). A WRKY transcription factor WRKY184 from *Brassica napus* L. @ is involved in flowering and secondary wall development in transgenic *Arabidopsis thaliana* . Plant Growth Regul. 92, 427–440. doi: 10.1007/s10725-020-00652-x

[B229] Yu D. Chen C. Chen Z. (2001). Evidence for an important role of WRKY DNA binding proteins in the regulation of NPR1 gene expression. Plant Cell 13, 1527–1540. doi: 10.1105/TPC.010115 11449049 PMC139550

[B230] Zarate S. I. Kempema L. A. Walling L. L. (2007). Silverleaf whitefly induces salicylic acid defenses and suppresses effectual jasmonic acid defenses. Plant Physiol. 143, 866–875. doi: 10.1104/pp.106.090035 17189328 PMC1803729

[B231] Zhang P. J. Shu J. P. Fu C. X. Zhou Y. Hu Y. Zalucki M. P.. (2008). Trade-offs between constitutive and induced resistance in wild crucifers shown by a natural, but not an artificial, elicitor. Oecologia 157, 83–92. doi: 10.1007/s00442-008-1060-8 18491145

[B232] Zhang P.-J. Li W. D. Huang F. Zhang J. M. Xu F. C. Lu Y. B. (2013). Feeding by whiteflies suppresses downstream jasmonic acid signaling by eliciting salicylic acid signaling. J. Chem. Ecol. 39, 612–619. doi: 10.1007/s10886-013-0283-2 23604702

[B233] Zhang L. Zhang F. Melotto M. Yao J. He S. Y. (2017). Jasmonate signaling and manipulation by pathogens and insects. J. Exp. Bot. 68, 1371–1385. doi: 10.1093/jxb/erw478 28069779 PMC6075518

[B234] Zhao L. Y. Chen J. L. Cheng D. F. Sun J. R. Liu Y. Tian Z. (2009). Biochemical and molecular characterizations of *Sitobion avenae*-induced wheat defense responses. Crop Prot. 28, 435–442. doi: 10.1016/j.cropro.2009.01.005

[B235] Zhao D. Luan Y. Shi W. Zhang X. Meng J. Tao J. (2021). A Paeonia ostii caffeoyl-CoA O-methyltransferase confers drought stress tolerance by promoting lignin synthesis and ROS scavenging. Plant Sci. 303, 110765. doi: 10.1016/j.plantsci.2020.110765 33487350

[B236] Zhou S. Jander G. (2022). Molecular ecology of plant volatiles in interactions with insect herbivores. J. Exp. Bot. 73, 449–462. doi: 10.1093/jxb/erab413 34581787

[B237] Zhou J. M. Zhang Y. (2020). Plant immunity: danger perception and signaling. Cell 181, 978–989. doi: 10.1016/j.cell.2020.04.028 32442407

[B238] Zhu J. Park K.-C. (2005). Methyl salicylate, a soybean aphid-induced plant volatile attractive to the predator *Coccinella septempunctata* . J. Chem. Ecol. 31, 1733–1746. doi: 10.1007/s10886-005-5923-8 16222805

[B239] Zukalová H. Vasak J. (2002). The role and effects of glucosinolates of Brassica species-a review. Rostlinna Vyroba 48, 175–180. doi: 10.17221/4217-PSE

