# Supplementary material for: Defense strategies and associated phytohormonal regulation in Brassica plants in response to chewing and sap-sucking insects
Source: Front Plant Sci. 2024 Apr 5;15:1376917. doi: 10.3389/fpls.2024.1376917 (PMC11026728; doi:10.3389/fpls.2024.1376917)
Supplement: Supplementary file 1 [file Table_1.docx]

**Supplementary Table S1. Showing the Effects of Insect Feeding on Physical and Chemical Defense Strategies in Brassica Plants**

| **Defense** | **Feeding style** | **Herbivore species** | **Plant species** | **Effect** | **References** |
| --- | --- | --- | --- | --- | --- |
| **Glucosinolates** | Sucking | *M. persicae*  *B. brassicae* | *A. thaliana* | Induction of indolic glucosinolate species (e.g., 4-methoxyindol-3-ylmethyl glucosinolate), gene expression associated with indole glucosinolate synthesis | (Mewis *et al.*, 2006; Kuśnierczyk *et al.*, 2007; Agerbirk *et al.*, 2009) |
|  | Sucking | *M. persicae*  *B. brassicae* | *A. thaliana* | Accumulation of glucosinolates | (Nouri-Ganbalani *et al.*, 2018) |
|  | Sucking | *L. erysimi* | *Brassica juncea-fruticulosa* introgression lines | Mixed results on glucosinolate content | (Palial, Kumar and Sharma, 2018) |
|  | Sucking | *L. erysimi* | *B. juncea* | Induction of transcripts related to biosynthetic pathways, attenuated by *L. erysimi* infestation | (Duhlian *et al.*, 2020) |
|  | Sucking | *L. erysimi*  *M. persicae* | *B. rapa* | Highest mean total glucosinolate content observed with *L. erysimi* infestation, lowest with *M. persicae* | (Blande, Pickett and Poppy, 2007) |
|  | Sucking | *B. brassicae* | *B. rapa* | Enhanced leaf growth and increased glucosinolate concentrations in the bulb | (Sotelo *et al.*, 2014) |
|  | Sucking | *M. persicae*, *B. brassicae* | *A. thaliana* | Different glucosinolate accumulation patterns, increased aliphatic content | (Mewis *et al.*, 2006) |
|  | Sucking | *B. brassicae* | *B. napus* | Induction of genes associated with the myrosinase-glucosinolate system | (Pontoppidan *et al.*, 2003) |
|  | Sucking | *B. brassicae* | *A. thaliana* | Consistent decrease in myrosinase transcript levels | (Kuśnierczyk *et al.*, 2007) |
|  | Chewing | *S. exigua* and *P. rapae* larvae | *A. thaliana* | Induction in both aliphatic and indole GSs, with a more pronounced effect on indole GS | (Mewis *et al.*, 2006; Textor and Gershenzon, 2009; Kos *et al.*, 2012; Gols *et al.*, 2018) |
|  | Chewing | *P. rapae* | *Brassica oleracea* | Increased foliar concentrations of glucosinolates compared to undamaged plants | (Broekgaarden *et al.*, 2007; Poelman *et al.*, 2008) |
|  | Chewing | *S. exigua* | *A. thaliana* | Increased aliphatic GS content | (Blande, Pickett and Poppy, 2007) |
|  | Chewing | *P. rapae* | *A. thaliana* | Slight increases in indole GS | (Blande, Pickett and Poppy, 2007; Kos *et al.*, 2012) |
|  | Chewing | *D. radicum* larvae | *B. rapa* (low and high GS varieties) | Upregulation of indole GS synthesis genes | (Sontowski *et al.*, 2019) |
|  | Chewing | *Mamestra brassicae, Autographa gamma, Pieris brassicae, Plutella xylostella* | *B. oleracea* | Increased in expression of indole GLS biosynthesis gene *CYP81F4* | (Karssemeijer *et al.*, 2022) |
|  |  |  |  |  |  |
| **Cell wall modification** | Sucking | *Brevicoryne brassicae* | *A. thaliana* | Induction of genes (WAK1, WAK2, At1g21245, At1g22720, and two (RFO1 and At1g79680) associated with cell wall | (Kuśnierczyk *et al.*, 2008) |
|  | Chewing | *P. rapae* | *A. thaliana* | Induced expression of AtMYB102, responsible for activation of genes responsible for cell wall remodeling | (De Vos *et al.*, 2006) |
| **Callose deposition** | Phloem feeding-sucking type | *Bemisia tabaci* | *A. thaliana* | Activation of Callose synthase 1 (*CALS1*), a gene responsible for callose deposition | (Kempema *et al.*, 2007) |
|  | Sucking | *Brevicoryne brassicae* | *A. thaliana* | Activation of Callose synthase 1 (*CALS1*), a gene responsible for callose deposition | (Kuśnierczyk *et al.*, 2008) |
| **Trichome** | Chewing | *P. rapae* | *B. rapa* | Increased trichome density | (Agren and Schemske, 1993) |
|  | Chewing | *P. rapae* | *B. nigra* | Increased trichome density | (Traw, 2002; Traw and Dawson, 2002) |
|  | Chewing | *Trichoplusia ni* | *B. nigra* | Increased trichome density | (Traw and Dawson, 2002), |
|  |  | *Spodoptera litura S. exigua* | *B. juncea* | Increased trichome density | (Mathur *et al.*, 2011). |
|  | Chewing | *P. rapae* | *Brassica nigra*, | Glabrous 1 (*GL1*) (a gene responsible for trichome development) | (Broekgaarden *et al.*, 2011). |
|  | Sucking | *B. brassicae* | *Brassica nigra* | Expression of O-methyltransferase family 2 protein, vitamin C defective 2 (*VTC2*), Xylogucan endotransglycosylase 6 (*XTH6*)), genes responsible for cell wall modification | (Broekgaarden *et al.*, 2011). |
